# Plasma exosome proteomics reveals the pathogenesis mechanism of post-stroke cognitive impairment

**DOI:** 10.18632/aging.204738

**Published:** 2023-05-20

**Authors:** Baoyun Qi, Lingbo Kong, Xinxing Lai, Linshuang Wang, Fei Liu, Weiwei Ji, Dongfeng Wei

**Affiliations:** 1 The Eastern Area, Dongzhimen Hospital, Beijing University of Chinese Medicine, Beijing 101121, China; 2 Institute of Basic Research in Clinical Medicine, China Academy of Chinese Medical Sciences, Beijing 100700, China; 3 Department of Neurology, Dongzhimen Hospital, Beijing University of Chinese Medicine, Beijing 100700, China; 4 Institute for Brain Disorders, Beijing University of Chinese Medicine, Beijing 100013, China; 5 Department of Neurology, Hohhot Mongolian Medicine of Traditional Chinese Medicine Hospital, Hohhot 010020, China; 6 Institute of Information on Traditional Chinese Medicine, China Academy of Chinese Medical Sciences, Beijing 100700, China

**Keywords:** blood flow regulation, lipid metabolism, plasma exosome, proteomics, post stroke cognitive impairment

## Abstract

Exploration and utilization of exosome biomarkers and their related functions provide the possibility for the diagnosis and treatment of post-stroke cognitive impairment (PSCI). To identify the new diagnostic and prognostic biomarkers of plasma exosome were uzed label-free quantitative proteomics and biological information analysis in PSCI patients. Behavioral assessments were performed, including the Mini-Mental Status Examination (MMSE), the Montreal Cognitive Assessment (MoCA), the Barthel index, the Morse Fall Seale (MFS) between control group (*n* = 10) and PSCI group (*n* = 10). The blood samples were collected to analyse the biomarker and differentially expressed proteins of plasma exosome using label-free quantitative proteomics and biological information. The exosomes marker proteins were determined by Western blot. The exosome morphology was observed by transmission electron microscopy. The scores of MMSE and MoCA were significantly decreased in the PSCI group. The PT% and high-density lipoprotein decreased and the INR ratio increased in PSCI group. The mean size of exosome was approximately 71.6 nm and the concentration was approximately 6.8E+7 particles/mL. Exosome proteomics identified 259 differentially expressed proteins. The mechanisms of cognitive impairment are related to regulate the degradation of ubiquitinated proteins, calcium dependent protein binding, cell adhesive protein binding, formation of fibrin clot, lipid metabolism and ATP-dependent degradation of ubiquitinated proteins in plasma exosome of PSCI patients. Plasma levels of YWHAZ and BAIAP2 were significantly increased while that of IGHD, ABCB6 and HSPD1 were significantly decreased in PSCI patients. These proteins might be target-related proteins and provide global insights into pathogenesis mechanisms of PSCI at plasma exosome proteins level.

## INTRODUCTION

Ischemic stroke is induced by cerebral artery occlusion, which causes damage to endothelial cells, vascular smooth muscle, glial cells, neurons and related neurovascular units, and ultimately leads to brain tissue death and focal neurological damage [1, 2]. Post-stroke cognitive impairment (PSCI) is a type of vascular cognitive impairment that manifests throughout 6 months following a stroke. Patients suffering from stroke lesions taking place in various regions of the brain that are not traditionally cognition-included may also result in development of PSCI. The plasma exosome biomarkers of PSCI have been emphasized. Plasma exosome biomarkers exploration and utilization and their associated functions allowed PSCI diagnosis and treatment possible.

Exosomes are small membrane vesicles existed in extracellular fluid and contain important genetic materials such as DNA, RNA, protein, lipid and miRNA [3]. Exosomes exist in extracellular fluid and contain important genetic materials such as DNA, RNA, protein, lipid and miRNA [3]. Exosomes can directly or indirectly act on target cells through the release of membrane contents or signal molecules for intercellular information transmission. In different pathological stages of ischemic stroke, exosomes released by different types of nerve cells contain specific signal molecules. Exosome miRNA-122-5p and miR-300-3p were biomarkers for the diagnosis of hyperacute (less than 6 h), subacute (8–14 d) and convalescent (greater than 14 d) ischemic cerebral infarction [4]. Exosomes secreted by circulating EPCs can transfer their inclusion to recipient endothelial cells, which contain miRNA related to PI3K/Akt signaling pathway and miRNA related to angiogenesis, such as miR-126 and miR-296. In the brain, exosomes secreted by cultured glioma cells provide angiogenic proteins, mRNAs and miRNAs to cerebrovascular endothelial cells and induce angiogenesis [5]. Neurons and glial cells interact with exosomes released by them to transfer biomolecules, regulate axonal growth and myelin sheath formation, and participate in brain nerve remodeling. Exosomes derived from multipluripotent mesenchymal stromal cells can effectively promote vascular and nerve regeneration, reduce inflammatory response and improve traumatic brain injury [6, 7]. Proteomics is an indispensable omics science to elucidate the proteome diversity. Faced with the limitations of diagnosis and treatment of PSCI, the task of finding effective methods to diagnose, predict and prevent PSCI has become more important.

In order to find a new prognostic and diagnostic plasma exosome biomarkers utilizing label-free quantitative proteomics and analysis of biological information in individuals with PSCI. In this study, a variety of psychological evaluations, which include the Mini-Mental Status Examination (MMSE), the Montreal Cognitive Assessment (MoCA), the Barthel index and blood biomarker detection were performed. The plasma exosome from control participants and PSCI patients were collected and analyzed by label-free quantitative proteomics. The differentially expressed proteins and its biological information analysis were conducted to establish global prospective on the PSCI pathogenesis mechanisms at proteins level.

## METHODS

### Study design

The Chinese Clinical Trial Registry (ChiCTR) has registered and recorded this clinical trial (registration number: ChiCTR1900023739, registration date: June 10, 2019), and the research protocol was approved by the Ethics Committee of Dongzhimen Hospital, a department of Beijing University of Chinese Medicine (approval number: DZMEC-KY-2019-04). Patients suffering from acute ischemic stroke were registered from Dongzhimen Hospital (eastern area) associated with Beijing University of Chinese Medicine between June 9, 2019 to December, 2019. The present investigation was conducted based on the ethical principles outlined in the Helsinki Declaration of 1975 (and as modified in 2013). This study contains the control group and PSCI group.

### Participants

This investigation included 20 participants who signed an informed consent form. There are 10 participants in the PSCI group and control group, respectively. PSCI patients’ inclusion criteria involved the following: (1) Age ≥35 years and ≤70 years; (2) Every patient has either an MRI or CT scan to validate the acute ischemic stroke; (3) Cognitive evaluation was done by MMSE within the first 7 days after the development of acute ischemic stroke. Patients having MMSE score ≤26 were determined as the PSCI group. (4) Cognitive impairment occurred after stroke [8, 9]. The exclusion criteria comprise the subsequent aspects : (1) Symptoms exhibited a range of complex to severe primary disorders of the heart, kidney, liver and hematopoietic system; (2) Consciousness acute disturbance; (3) Dementia and brain, different causes are because of mental or physiological diseases; (4) With severe vision, hearing or even speech impairment conflicts with rehabilitation; and (5) Furthermore the onset of cognitive impairment, there were no additional focal indications of cerebrovascular disease.

### Behavioral assessment

The informed consent forms were signed by all participants and a variety of behavioral evaluations were conducted, such as the MMSE, the MoCA, the Barthel index, the Morse Fall Seale (MFS), and The Braden Scale is a scale utilised for the prediction of pressure sore risk, as well as the Padua Prediction Score. The patients received antiplatelet therapy (aspirin, 100 mg, QD) before assessments.

### Blood biomarker detection

After a fasting period of 12-h, blood samples were obtained in the morning. Two milliliters of whole blood were drawn from peripheral vein of each participant and kept in a polypropylene tube including EDTA. The Dongzhimen Hospital affiliated with Beijing University of Chinese Medicine was requested to furnish the blood specimens. Following that, the fibrinogen, red blood cell specific volume (HCT), total cholesterol (CHO), triglyceride (TG), high-density lipoprotein (HDL), low-density lipoprotein (LDL), and other different markers were measured using whole blood samples.

### The isolation and determination of plasma exosomes characteristics

#### The isolation procedure of exosomes

The isolation of plasma exosomes using TiO2 with a slight modification [10]. Remove the plasma sample from storage and place it on ice. Centrifuge the plasma sample at 2000 × g at room temperature for 20 min to extract cells and debris. Transfer 100 μL plasma to a new tube and processed using 0.2 mm pore size syringe filters (PALL Life Sciences, USA) for extracting apoptotic bodies and the large microvesicles. Following that, 5 mg of TiO2 microspheres were combined with the plasma sample (GL Science Inc, Japan) and mixed the sample thoroughly by vortexing at 4°C for a period of 5 min. The mixture underwent centrifugation at a force of 20,000 × g for 3 min at a temperature of 4°C, subsequently, the supernatant was extracted. The exosomes on the TiO2 microspheres were hydrated with PBS three times to eliminate unspecific contaminants. After washing with PBS, exosomes were lysed and digested directly with trypsin (Promega, Madison, WI) from the surface of microspheres at 37°C in 50 mM ammonium bicarbonate for 16 h. A new tube was used to transfer the supernatant and the TiO2 microspheres were hydrated twice with 100 μL of 0.1% formic acid after centrifugation at 12,000 g at 25°C for 5 min. The washing fraction was extracted and pooled with the supernatant. NanoDrop (Thermo Fisher, USA) was utilized to measure the concentration of peptide at an absorbance of 280 nm.

#### Western blot analysis of exosomes marker proteins

Detect the quality of plasma protein in 0.5 mL fraction sample. The fraction of vesicles with the least plasma protein content was selected for subsequent analysis. The concentration of protein samples was measured utilizing the BCA method. 12% SDS-PAGE was used for separating 10 μg of the protein, then the protein transferred to a 0.45 μm PVDF membrane, and inhibited utilizing a blocking solution including 5% bovine serum albumin for a period of 1 h at room temperature. Exosomal marker protein antibodies were add and incubate at a temperature of 4°C overnight, containing anti-CD63 rabbit polyclonal antibody (1:600) (Cat No. 25682-1-AP, Proteintech Group, Chicago, IL, USA), anti-TSG101 rabbit polyclonal antibody (1:2000) (Cat No. 28283-1-AP, Proteintech Group, USA), anti-CD81 mouse polyclonal antibody (1:3500) (Cat No. 66866-1-Ig, Proteintech Group, USA), anti-CD9 mouse polyclonal antibody (1:3000) (Cat No. 20597-1-AP, Proteintech Group, USA) and HRP-conjugated secondary antibodies (1:6000) (Cat No. SA00001-2; Proteintech Group, USA). The western blotting was examined utilizing an eECL Western blot kit (Cat No. CW0049 M, CWBIO, Jiangsu, China). After being eluted with 1 × TBST buffer, secondary antibody was supplemented and incubated at room temperature for a period of 90 min. After 3 hydrations by TBST, the color was generated utilizing SuperSignal West Femto Substrate Trial Kit (34094, Pierce, Rockford, IL, USA).

#### Transmission electron microscopy (TEM)

The exosome morphology was observed utilizing TEM. Exosome sample drops have the ability to adsorb for 5 min on formvar-coated EM grids, and were stained negatively utilizing 2% (w/v) phosphotungstic acid for 1 min. At 80 kV of acceleration voltage, transmission electron microscope (H-7650; Hitachi, Ltd., Tokyo, Japan) was utilized to perform TEM analysis.

#### Nanoparticle tracking analyzer (NTA) and Dynamic light scattering (DLS) analysis

The particle size and concentration analysis of model exosomes were performed on ZetaView Nanoparticle Tracker (Particle Metrix, Meerbusch, Germany). Calibrate the instrument with polystyrene particles with approximately 100 nm of a particle size, Dilute the model exosomes to approximately 1 × 10^8^ particles/mL, and put them into the instrument for analysis. Each group of samples is automatically scanned 11 times to remove abnormal data. The data was recorded and analyzed by ZetaView 8.03.04.01 software.

### Label-free quantitative proteomics

MALDI-TOF-MS/MS and database searching were utilized for the identification of proteins. An online liquid chromatography-tandem mass spectrometry (LC-MS/MS) setup including an EasynanoLC system and a Q-Exactive mass spectrometer (Thermo Scientific, Germany) installed with a nanoelectrospray ion source was utilized for all LC-MS/MS investigations.

(1) Mobile phase A included 0.1% FA, 2% acetonitrile dissolved within water, and mobile phase B included 0.1% FA, 98% acetonitrile in water. The flow rate has been measured to be 300 nL/min.

(2) At 2 kV, the source was operated. In order to perform a full MS survey scan, AGC target was 3e6, scan range was from m/z 300 to 1400 and the result of resolution was 70,000. The 50 highest intense peaks with charge state 2 and above were chosen in order to sequence and fragmented in the ion trap by HCD with normalized collision energy of 27%. Exclude isotope item was enabled and dynamic exclusion time was adjusted as 18 s.

(3) MaxQuant software was utilized to search the Raw MS files against UniProt database. (Version 1.5.2.8). The fixed modification was C (carbamidomethyl) and the variable modification was M (oxidation) and protein N-term (acetyl). The tolerance for the first search peptide was 20 ppm and the tolerance for the main search peptide was 6 ppm. The MS/MS tolerance result was 0.02Da. The PSM and protein false discovery level result was 1%. The employed among the runs and minimum score required for modified peptides was 40.

### Biological information analysis

#### Protein-protein interaction (PPI) networks analysis

To gain a deeper comprehension from the perspective of the biological context of differentially expressed proteins, the protein interaction analysis was conducted by utilizing the free web-based search tool STRING10.5. The STRING database is a fundamental data resource within the ELIXIR’s core, containing both identified and anticipated protein interactions. These data will be collected and integrated by the STRING database, with consolidating identified and anticipated protein-protein correlation data for the organism’s large number [11]. STRING was needed to incorporate additional predicted functional partners for the purpose of improving the PPI networks formation. The protein IDs list that was differentially expressed was entered into the STRING database (https://string-db.org) to determine identified and anticipated protein functional correlation networks.

#### GO analysis

For exhibiting the differentially expressed proteins presence, to categorize the proteins in accordance with their biological process, protein classification, cellular composition, and pathway, GO enrichment analysis was conducted. In order to find out how these experimentally discovered proteins are distributed, GO enrichment annotation tools were used to examine each protein with higher speed and to comprehend the relationship between protein and biological function in its entirety. GO function enrichment analysis of protein describes this distribution comparison with the overall protein distribution, confirming which biological processes or molecular functions were significantly enriched with experimentally identified proteins, many regulatory, metabolic, and signal transduction pathways are resistant within the organism, and these pathways frequently form various pathways. Pathway analysis permits the identification of the highest significant biochemical-metabolic and signal transduction pathways in which the proteins were contained.

### ELISA quantification analysis

To detect the levels of IGHD, ABCB6, HSPD1, YWHAZ and BAIAP2, enzyme-linked immunosorbent assay (ELISA) kits were utilized to determine the plasma. According to the manufacturer guidelines, plasma samples were thawed and submitted to ELISA quantitative analysis using human IgD ELISA kit (Abcam, ab157708, Cambridge, UK), human ATP Binding Cassette Subfamily B Member 6, Mitochondrial (ABCB6) ELISA Kit (Abbexa, abx385541, Cambridge, UK), human Heat Shock Protein 60, HSP-60 ELISA Kit (CSB-E08560h, Cusabio, China), human 14-3-3 protein zeta/delta (YWHAZ) ELISA kit (CSB-EL026293HU, Cusabio, China), human Brain-Specific Angiogenesis Inhibitor 1-Associated Protein 2 (BAIAP2) ELISA Kit (Abbexa, abx386001, Cambridge, UK). ELISA microplates were read using MK3 microplate reader (Thermo, Helsinki, Finland).

### Statistical analysis

Each value was reported as the mean ± standard deviation (SD). Statistical analysis was conducted by SPSS20.0. The Demographic data and MMSE, MoCA, blood marker were analyzed with independent-samples *t*-test between control group and PSCI group. The Chi-square statistical test was utilized to investigate potential differences in gender variation between two distinct groups. *P*-values < 0.05 were reported as statistically significant.

## RESULTS

### Demographics, behavioral assessment and detection of blood biomarker results

There were no variations in age (*P* = 0.182), gender (*P* = 0.653) or education (*P* = 0.072) among the PSCI and the control groups. The MMSE and MoCA scores were significantly reduced throughout the PSCI groups (*P* < 0.01) (Table 1). According to the control group, the PT% and high-density lipoprotein reduced (*P* < 0.05) and the INR ratio reduced (*P* < 0.05) in the PSCI group (Table 2).

**Table 1:** Demographic information and behavioral scale assessment.

	**Control group (*n* = 10)**	**PSCI group (*n* = 10)**	***P*-value**
Age (y)	55.80 ± 6.92	60.30 ± 9.17	0.231
Gender (M/F)	6/4	5/5	0.653
Education (y)	10.30 ± 2.16	8.30 ± 2.50	0.072
MMSE	29.80 ± 0.42	20.30 ± 3.83	<0.01
MoCA	29.40 ± 0.70	15.70 ± 5.03	<0.01
Barthel	–	70.00 ± 15.28	–
Morse Fall Seale	–	46.50 ± 10.81	–
Braden Scale	–	20.50 ± 2.32	–
Padua Prediction Score	–	2.70 ± 2.06	–

Abbreviations: MMSE: Mini-Mental Status Examination; MoCA: Montreal Cognitive Assessment; Barthel: The Barthel index and data are presented as mean ± SD or number of patients.

**Table 2:** The blood marker detection results.

	**Control group (*n* = 10)**	**PSCI group (*n* = 10)**	***P*-value**
**Blood coagulation function**			
PT%	102.13 ± 9.67	91.90 ± 7.58	0.027^*^
INR ratio	1.00 ± 0.05	1.06 ± 0.06	0.045^*^
Activated partial thromboplastin time	30.14 ± 3.99	31.33 ± 5.19	0.619
Fibrinogen content	2.61 ± 0.26	2.80 ± 0.62	0.470
Thrombin time	15.37 ± 1.16	14.86 ± 1.33	0.424
D-dimer	62.43 ± 27.65	118.6 ± 73.42	0.075
**Blood biochemistry**			
Serum creatinine	61.40 ± 14.89	60.60 ± 21.26	0.923
Glucose	6.54 ± 2.35	8.87 ± 3.68	0.109
Albumin	39.07 ± 4.57	38.44 ± 4.52	0.760
Calcium	2.29 ± 0.09	2.34 ± 0.13	0.368
Total bilirubin	13.69 ± 3.55	19.93 ± 8.57	0.047^*^
Direct bilirubin	3.23 ± 1.10	4.54 ± 2.47	0.143
Indirect bilirubin	10.46 ± 3.32	15.39 ± 6.43	0.045^*^
Alanine aminotransferase	17.30 ± 6.45	24.90 ± 13.16	0.118
Aspartate aminotransferase	19.40 ± 4.27	34.40 ± 35.08	0.196
Lactate dehydrogenase	152.67 ± 29.82	210.90 ± 47.47	0.006^**^
Hydroxybutyric acid dehydrogenase	114.33 ± 22.87	154.50 ± 40.21	0.017^*^
γ-glutamyltransferase	17.70 ± 6.63	29.50 ± 18.85	0.078
Alkaline phosphatase	60.89 ± 8.62	69.70 ± 20.31	0.245
Adenylate dehydrogenase	10.00 ± 2.87	10.70 ± 5.44	0.735
**Serum lipid**			
Total cholesterol	4.38 ± 0.56	4.18 ± 1.46	0.691
Apolipoprotein A1	1.17 ± 0.11	1.10 ± 0.17	0.401
Apolipoprotein B	0.83 ± 0.23	0.74 ± 0.26	0.501
High-density lipoprotein	0.98 ± 0.15	0.84 ± 0.13	0.045^*^
Low density lipoprotein	2.62 ± 0.55	2.42 ± 1.25	0.662
Lipoprotein (a)	182.00 ± 115.42	267.78 ± 176.09	0.285
Homocysteine	13.63 ± 3.75	15.78 ± 7.62	0.512

Values are mean ± SD. ^*^P < 0.05, ^**^P < 0.01.

### Isolation and characterization results of plasma exosomes

**Figure 1:**
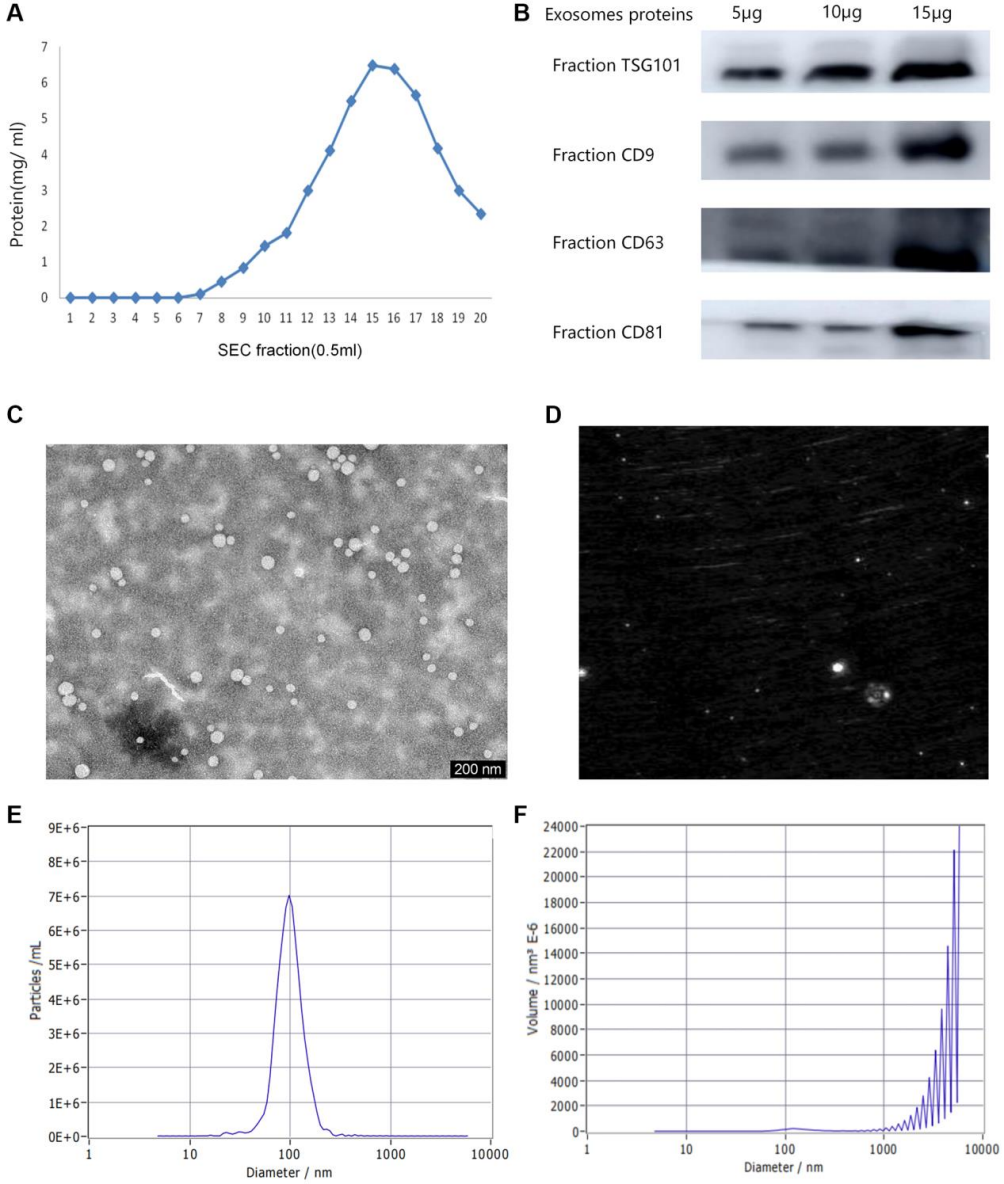
**Various characterizations of plasma exosomes.** (**A**) The content of plasma proteins in 0.5 mL fraction sample. (**B**) Western blot analysis of the typical exosomal proteins, TSG 101, CD9, CD63 and CD81. (**C**) Transmission electron microscopy (TEM) image indicating exosome morphology. (**D**) A representative laser scattering microscopy image of isolated exosomes. (**E**) The Nanoparticle tracking analysis (NTA) result of the particle size distribution for isolated exosomes. (**F**) The size distribution of volume consistent with the size range of exosomes.

The content detection results of plasma protein in 0.5 mL fraction sample were shown in Figure 1A. The content of plasma protein in fractions 6–10 is relatively lower, and the purity of exosomes is relatively high. Therefore, the 5 vesicle fractions 6–10 were selected for subsequent analysis. Western blot analysis was utilized to detect the exosomal protein markers in all the plasma exosome samples. The exosomal markers of protein expression levels (TSG101, CD9, CD63, and CD81) were shown in Figure 1B. The exosome morphology and size were visualized by TEM (Figure 1C). TEM observation revealed highly homogenous exosome combination with a regular round morphology having a diameter range of 30–200 nm. A representative laser scattering microscopy image of isolated exosomes is shown in Figure 1D. NTA was exploited to measure the size distribution of particles and view the isolated vesicle samples (Figure 1E, 1F). The samples fluorescent detection examined in the scatter mode. The size is calculated by the diffusion behavior. The study determined that the mean size of the particles was 71.6 nm, while the concentration was estimated to be around 6.8E+7 particles/mL.

### Differentially expressed exosome proteins determination among the control and PSCI groups

**Figure 2:**
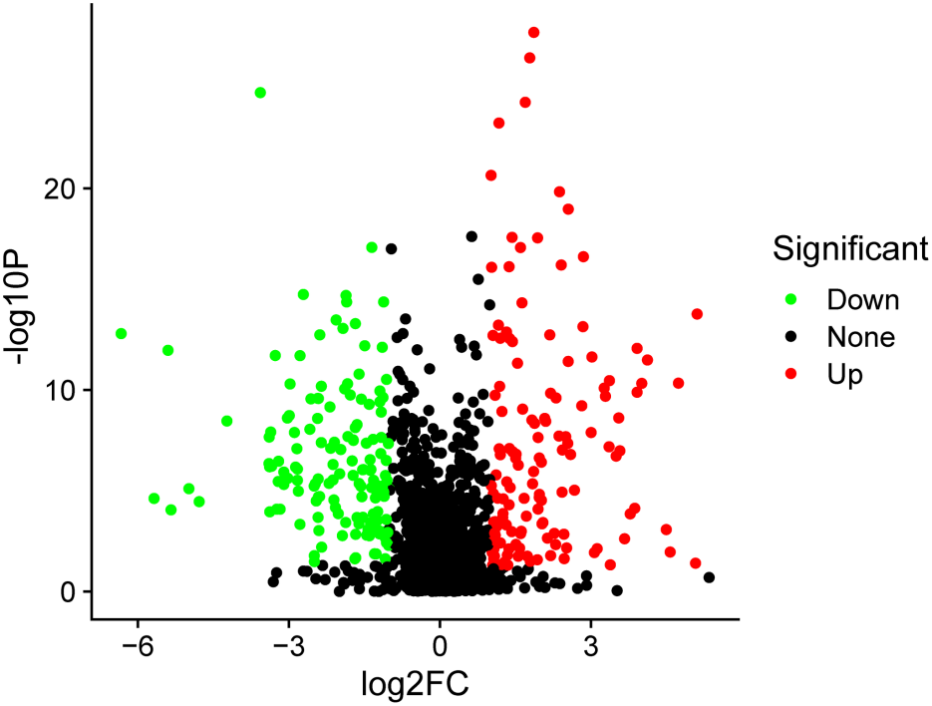
**The volcano plot of differentially expressed proteins.** The red points represented up-regulated proteins and green points represented down-regulated proteins between the PSCI and control groups.

**Figure 3:**
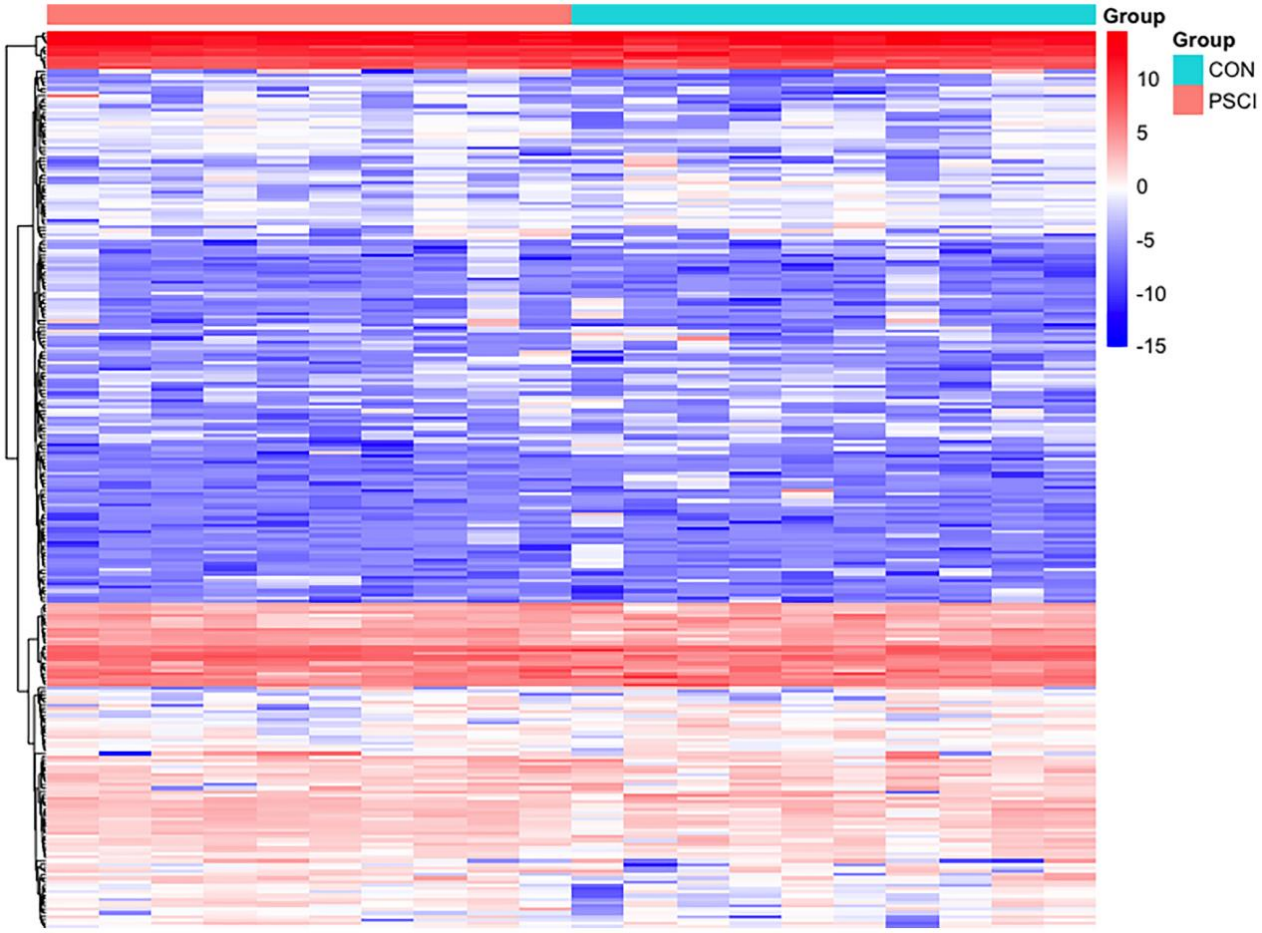
**Hierarchical clustering of plasma exosome proteomes.** The heat map represented the Z scores of all proteins quantified in Label-free quantitative proteomics.

In total, 259 differentially expressed exosome proteins have been measured and determined between the control group and PSCI group by Label-free quantitative proteomics, of which 131 proteins demonstrated up-regulated expression and 128 proteins showed down-regulated expression. The differentially expressed proteins with PSCI/control ratios that are high or less than 1.2-fold change having a set *P*-value < 0.05 were shown to be significantly changed. The differentially expressed proteins volcano plot was presented in Figure 2. The heat map of the whole differential expressed proteins were presented in Figure 3. The identification results of main 30 up-regulated proteins were presented in Table 3 and the other 101 up-regulated proteins were shown in Supplementary Table 1. The identification results of main 30 down-regulated proteins were shown in Table 4 and the other 98 down-regulated proteins were shown in Supplementary Table 2. The results of these proteins are summarized in detail. According to Table 5, the biological process category of 30 up-regulated proteins and molecular function were conducted according to biological function. The biological process category of 27 down-regulated proteins and molecular function were shown in Table 6.

**Table 3:** The identification results of main 30 up-regulated proteins between the PSCI group and control group.

**No.**	**Accession No.**	**Protein name**	**Symbol**	**Exp. Mr (KDa)**	**Protein score**	**Fold change (PSCI/Control)**	***P*-value**
1	P63104	14-3-3 protein zeta/delta	YWHAZ	27.75	177.81	2.13	<0.001
2	P31947	14-3-3 protein sigma	SFN	27.77	8.19	3.04	<0.01
3	Q9UQB8	Brain-specific angiogenesis inhibitor 1-associated protein 2	BAIAP2	60.87	43.47	4.95	<0.01
4	P78417	Glutathione S-transferase omega-1	GSTO1	25.90	27.57	3.00	<0.01
5	P61421	V-type proton ATPase subunit d 1	ATP6V0D1	40.33	8.42	2.91	<0.01
6	P34932	Heat shock 70 kDa protein 4	HSPA4	94.33	7.37	7.05	<0.01
7	P40197	Platelet glycoprotein V	GP5	60.96	49.41	5.19	<0.01
8	P37235	Hippocalcin-like protein 1	HPCAL1	22.31	5.46	5.82	
9	Q9Y6B6	GTP-binding protein SAR1b	SAR1B	22.41	5.08	2.28	<0.01
10	P54920	Alpha-soluble NSF attachment protein	NAPA	17.26	33.23	34.53	<0.01
11	P13746	Class I histocompatibility antigen, A-11 alpha chain	HLA-A	15.44	40.94	33.79	<0.05
12	P51575	P2X purinoceptor 1	P2RX1	13.33	44.98	15.11	<0.01
13	O76074	cGMP-specific 3,5-cyclic phosphodiesterase	PDE5A	17.60	99.98	11.74	<0.01
14	Q13642	Four and a half LIM domains protein 1	FHL1	16.00	36.26	8.72	<0.01
15	Q9BQE5	Apolipoprotein L2	APOL2	2.9676	37.092	5.85	<0.01
16	P08648	Integrin alpha-5	ITGA5	10.36	114.54	5.73	<0.01
17	O94804	Serine/threonine-protein kinase 10	STK10	11.78	112.13	3.86	<0.01
18	P11169	Solute carrier family 2, facilitated glucose transporter member 3	SLC2A3	76.41	53.92	3.65	<0.01
19	Q15762	CD226 antigen	CD226	23.75	38.61	3.45	<0.01
20	Q93084	Sarcoplasmic/endoplasmic reticulum calcium ATPase 3	ATP2A3	17.16	113.98	3.04	<0.01
21	O00194	Ras-related protein Rab-27B	RAB27B	21.19	24.61	2.69	<0.01
22	Q9NP79	Vacuolar protein sorting- associated protein VTA1 homolog	VTA1	28.89	33.88	2.63	<0.01
23	P14770	Platelet glycoprotein IX	GP9	71.37	19.05	2.61	<0.01
24	Q9UN37	Vacuolar protein sorting- associated protein 4A	VPS4A	11.56	48.897	2.37	<0.001
25	Q08830	Fibrinogen-like protein 1	FGL1	2.9253	36.379	9.61	<0.001
26	P02763	Alpha-1-acid glycoprotein 1	ORM1	13.49	23.51	2.12	<0.01
27	P02749	Beta-2-glycoprotein 1	APOH	233.67	38.30	2.11	<0.01
28	P55058	Phospholipid transfer protein	PLTP	20.08	54.74	2.10	<0.01
29	P30273	High affinity immunoglobulin epsilon receptor subunit gamma	FCER1G	10.82	9.67	2.04	<0.01
30	P01624	Ig kappa chain V-III region POM	IGKV3-15	17.61	11.92	2.03	<0.01

In the title line, Exp. Mr represented the experimental molecular weight of the proteins.

**Table 4:** The identification results of main 30 down-regulated proteins between the PSCI group and control group.

**No.**	**Accession No.**	**Target protein**	**Symbol**	**Exp. Mr (KDa)**	**Protein score**	**Fold change (PSCI/Control)**	***P*-value**
1	P06276	Cholinesterase	BCHE	12.41	68.42	0.41	<0.05
2	P01880	Ig delta chain C region	IGHD	42.25	17.46	0.02	<0.01
3	P04438	Ig heavy chain V-II region SESS		16.32	4.15	0.18	<0.01
4	P01771	Ig heavy chain V-III region HIL		13.44	39.48	0.27	<0.01
5	P01778	Ig heavy chain V-III region ZAP		12.34	6.03	0.10	<0.01
6	P07357	Complement component C8 alpha chain	C8A	65.16	22.61	0.14	<0.01
7	P20618	Proteasome subunit beta type-1	PSMB1	26.49	12.65	0.11	<0.01
8	Q9P289	Serine/threonine-protein kinase 26	STK26	46.53	2.66	0.13	<0.01
9	Q99536	Synaptic vesicle membrane protein VAT-1 homolog	VAT1	8.1909	41.92	0.48	<0.01
10	P03952	Plasma kallikrein	KLKB1	71.37	7.30	0.20	<0.01
11	P53396	ATP-citrate synthase	ACLY	120.84	21.57	0.41	<0.01
12	Q9NP58	ATP-binding cassette sub-family B member 6, mitochondrial	ABCB6	93.88	4.74	0.24	<0.01
13	P62330	ADP-ribosylation factor 6	ARF6	20.08	11.40	0.26	<0.05
14	P10809	60 kDa heat shock protein, mitochondrial	HSPD1	61.05	88.42	0.47	<0.01
15	O15162	Phospholipid scramblase 1	PLSCR1	35.05	10.23	0.11	<0.01
16	Q99828	Calcium and integrin-binding protein 1	CIB1	21.70	8.13	0.11	<0.01
17	Q5D862	Filaggrin-2	FLG2	248.07	81.40	0.12	<0.01
18	Q8WXI7	Mucin-16	MUC16	2284.30	8.38	0.13	<0.01
19	P56199	Integrin alpha-1	CFHR1	130.85	4.11	0.14	<0.01
20	P25311	Zinc-alpha-2-glycoprotein	AZGP1	34.26	8.18	0.15	<0.01
21	P02545	Prelamin-A/C	LMNA	74.14	26.39	0.01	<0.01
22	P30486	Class I histocompatibility antigen, B-48 alpha chain	HLA-B	40.36	29.55	0.03	<0.01
23	Q9NP59	Solute carrier family 40-member 1	SLC40A1	62.54	9.51	0.10	<0.01
24	P13164	Interferon-induced transmembrane protein 1	IFITM1	13.96	6.85	0.10	<0.01
25	O14818	Proteasome subunit alpha type-7	PSMA7	27.89	17.47	0.17	<0.01
26	P22234	Multifunctional protein ADE2	PAICS	47.08	10.28	0.17	<0.01
27	P30153	Serine/threonine-protein phosphatase 2A 65 kDa regulatory subunit A alpha isoform	PPP2R1A	65.31	10.92	0.18	<0.01
28	Q9HCM2	Plexin-A4	PLXNA4	212.45	5.19	0.18	<0.05
29	Q8NG06	E3 ubiquitin-protein ligase TRIM58	TRIM58	54.77	29.55	0.18	<0.05
30	Q9BS26	Endoplasmic reticulum resident protein 44	ERP44	46.97	3.45	0.19	<0.01

In the title line, Exp. Mr represented the experimental molecular weight of the proteins.

**Table 5:** The molecular function and biological process category of main 30 up-regulated expressed proteins in PSCI.

**No.**	**Target protein**	**Molecular function**	**Biological process**
1	14-3-3 protein zeta/delta	Cadherin binding; ion channel binding; protein domain specific binding; protein kinase binding	Establishment of Golgi localization; protein insertion into mitochondrial membrane involved in apoptotic signaling pathway; synaptic target recognition
2	14-3-3 protein sigma	Cadherin and phosphoprotein binding; protein kinase C inhibitor activity	Intrinsic apoptotic signaling pathway; regulation of protein insertion into mitochondrial membrane involved in apoptotic signaling pathway; release of cytochrome c from mitochondria
3	Brain-specific angiogenesis inhibitor 1-associated protein 2	Cadherin binding involved in cell-cell adhesion; identical protein binding; proline-rich region binding; scaffold protein binding; transcription cofactor binding	Axonogenesis; cellular response to L-glutamate; modification of synaptic structure, modulating synaptic transmission; regulation of synaptic plasticity; vascular endothelial growth factor receptor signaling pathway
4	Glutathione S-transferase omega-1	Glutathione dehydrogenase (ascorbate) activity; glutathione transferase activity; methylarsonate reductase activity; oxidoreductase activity	L-ascorbic acid metabolic process; cellular response to arsenic-containing substance; glutathione derivative biosynthetic process; interleukin-12 mediated signaling pathway
5	V-type proton ATPase subunit d 1	Proton-exporting ATPase activity, phosphorylative mechanism proton-transporting ATPase activity	IRE1-mediated unfolded protein response; cellular iron ion homeostasis; cellular response to increased oxygen levels; phagosome acidification; proton transmembrane transport
6	Heat shock 70 kDa protein 4	ATP binding	Chaperone-mediated protein complex assembly; protein insertion into mitochondrial outer membrane; response to unfolded protein
7	Platelet glycoprotein V	Mediates vWF-dependent platelet adhesion to blood vessels	Blood coagulation, intrinsic pathway; cell adhesion; platelet activation
8	Hippocalcin-like protein 1	Calcium ion binding	
9	GTP-binding protein SAR1b	GTP binding; GTPase activity; metal ion binding	Antigen processing and presentation of exogenous peptide antigen via MHC class I and II; endoplasmic reticulum to Golgi vesicle-mediated transport; intracellular protein transport;
10	Alpha-soluble NSF attachment protein	Protein containing complex binding; soluble NSF attachment protein activity; syntaxin binding	Endoplasmic reticulum to Golgi vesicle mediated transport; synaptic vesicle priming; synaptic transmission, glutamatergic
11	P2X purinoceptor 1	ATP binding; ATP-gated ion channel activity; extracellularly ATP-gated cation channel activity; purinergic nucleotide receptor activity; zinc ion binding	Apoptotic process; calcium ion transport; neuronal action potential; Regulation of presynaptic cytosolic calcium ion concentration; synaptic transmission
12	cGMP-specific 3,5-cyclic phosphodiesterase	3′,5′-cyclic-GMP phosphodiesterase activity; cGMP binding; metal ion binding	cGMP catabolic process; MAP kinase activity; regulation of nitric oxide mediated signal transduction
13	Four and a half LIM domains protein 1	Ion channel binding; metal ion binding	Cell differentiation; potassium ion transport; membrane depolarization; regulation of potassium ion transmembrane transporter activity
14	Apolipoprotein L2	High density lipoprotein particle binding; lipid binding; signaling receptor binding	Cholesterol metabolic process; lipid metabolic process; lipid transport; lipoprotein metabolic process
15	Tetraspanin-32	Cytoskeleton organization	Cell-cell signaling; defense response to protozoan; integrin mediated signaling pathway; platelet aggregation;
16	Integrin alpha-5	Epidermal growth factor receptor binding; metal ion binding; platelet derived growth factor receptor binding; vascular endothelial growth factor receptor 2 binding	Angiogenesis; cell adhesion; cell substrate junction assembly; endodermal cell differentiation; cell migration; peptidyl tyrosine phosphorylation;
17	Serine/threonine-protein kinase 10	ATP binding; identical protein binding; protein homodimerization activity; protein serine/threonine kinase activity	Activation of protein kinase activity; lymphocyte aggregation and migration; neutrophil degranulation; protein autophosphorylation;
18	Solute carrier family 2, facilitated glucose transporter member 3	Glucose binding; glucose transmembrane transporter activity	L-ascorbic acid metabolic process; carbohydrate metabolic process; glucose transmembrane transport; neutrophil degranulation
19	CD226 antigen	Cell adhesion molecule binding; integrin binding; protein kinase binding	Cell recognition; cytokine production; T cell receptor signaling pathway; immunoglobulin mediated immune response; interferon-gamma production
20	Sarcoplasmic/endoplasmic reticulum calcium ATPase 3	ATP binding; calcium transmembrane transporter activity; metal ion binding; proton exporting ATPase activity	Calcium ion transmembrane transport; calcium ion transport; cellular calcium ion homeostasis; ion transmembrane transport
21	Ras-related protein Rab-27B	GDP binding; GTP binding; GTPase activity; myosin V binding; protein domain specific binding	Rab protein signal transduction; anterograde axonal protein transport; intracellular protein transport; multivesicular body sorting pathway; synaptic vesicle endocytosis
22	Vacuolar protein sorting-associated protein VTA1 homolog	Protein C-terminus binding	ESCRT III complex disassembly; endosomal transport; macroautophagy; multivesicular body assembly; multivesicular body sorting pathway
23	Platelet glycoprotein IX	Platelet activation apparently involves disruption of the macromolecular complex of GP-Ib with the platelet glycoprotein IX	Blood coagulation, intrinsic pathway; cell adhesion; platelet activation
24	Vacuolar protein sorting-associated protein 4A	ATP binding; ATPase activity; protein C-terminus binding; protein domain specific binding; protein containing complex binding	Ubiquitin-dependent protein catabolic process via the multivesicular body sorting pathway; exosomal secretion; multivesicular body assembly
25	Fibrinogen-like protein 1	Inhibiting inflammatory immune responses and metabolic function	Adaptive immune response
26	Alpha-1-acid glycoprotein 1	Functions as transport protein in the blood stream	Inflammatory response; neutrophil degranulation; platelet degranulation; interleukin-1 beta secretion
27	Beta-2-glycoprotein 1	Heparin binding; lipid binding; lipoprotein lipase activator activity; phospholipid binding	Angiogenesis; plasminogen activation; platelet degranulation; blood coagulation; regulation of fibrinolysis; triglyceride metabolic process
28	Phospholipid transfer protein	Ceramide binding and transfer activity; lipid transporter activity; phosphatidic acid binding and transfer activity;	Ceramide transport; high-density lipoprotein particle remodeling; lipid metabolic process; phospholipid transport; cholesterol efflux; vitamin E biosynthetic process
29	High affinity immunoglobulin epsilon receptor subunit gamma	IgE binding, IgE receptor activity, IgG binding, protein homodimerization activity	Immunoglobulin mediated immune response, innate immune response, neutrophil chemotaxis, T cell differentiation
30	Ig kappa chain V-III region POM	Antigen binding	Fc-gamma receptor signaling pathway involved in phagocytosis; complement activation, classical pathway; immune response; leukocyte migration;

**Table 6:** The molecular function and biological process category of main 27 down-regulated proteins in PSCI.

**No.**	**Target protein**	**Molecular function**	**Biological process**
1	Cholinesterase	Acetylcholinesterase activity; amyloid-beta binding; choline binding; cholinesterase activity; enzyme binding	Choline metabolic process; cocaine metabolic process; neuroblast differentiation; response to alkaloid and folic acid; response to glucocorticoid
2	Ig delta chain C region	Antigen binding; immunoglobulin receptor binding	B cell receptor signaling pathway; complement activation, classical pathway; innate immune response; positive regulation of interleukin-1 secretion
3	Complement component C8 alpha chain	Complement binding; protein-containing complex binding	Complement activation, alternative or classical pathway; immune response; regulation of complement activation
4	Proteasome subunit beta type-1	Endopeptidase activity; threonine type endopeptidase activity	Proteasomal ubiquitin dependent protein catabolic process; Wnt signaling pathway; post translational protein modification; protein polyubiquitination;
5	Serine/threonine-protein kinase 26	ATP binding; magnesium ion binding; protein homodimerization activity; protein kinase activity; protein serine/threonine kinase activity	Activation of protein kinase activity; neuron projection morphogenesis; protein phosphorylation; signal transduction by protein phosphorylation
6	Angiopoietin-like protein 8	Hormone activity	cEll maturation; cellular lipid metabolic process; lipid metabolic process; lipoprotein lipase activity;
7	Plasma kallikrein	Serine-type endopeptidase activity	Factor XII activation; blood coagulation, intrinsic pathway; extracellular matrix disassembly; fibrinolysis; plasminogen activation
8	ATP-citrate synthase	ATP binding; ATP citrate synthase activity; cofactor binding; metal ion binding	Acetyl-CoA and cholesterol biosynthetic process; citrate metabolic process; coenzyme A metabolic process
9	ATP-binding cassette sub-family B member 6, mitochondrial	ATP binding; ATPase activity; ATPase-coupled heme transmembrane transporter activity; heme binding	Cellular iron ion homeostasis; heme transport; transmembrane transport
10	ADP-ribosylation factor 6	GTP binding; GTPase activity; protein N-terminus binding; thioesterase binding	Intracellular protein transport; maintenance of postsynaptic density structure; protein localization to endosome; synaptic vesicle endocytosis
11	60 kDa heat shock protein, mitochondrial	ATP binding; ATPase activity; apolipoprotein binding; chaperone binding	Chaperone-mediated protein complex assembly; B cell activation and cytokine production; protein import into mitochondrial intermembrane space
12	Phospholipid scramblase 1	CD4 receptor binding; DNA binding transcription activator activity, calcium ion binding; phospholipid scramblase activity	Apoptotic process; phosphatidylserine biosynthetic process; plasma membrane phospholipid scrambling
13	Calcium and integrin-binding protein 1	Ras GTPase binding; calcium ion binding; calcium dependent protein kinase inhibitor activity; protein C-terminus binding; protein kinase binding	Angiogenesis; apoptotic process; cell adhesion; cellular response to DNA damage stimulus and growth factor stimulus; cytoplasmic microtubule organization
14	Filaggrin-2	Calcium ion binding; structural constituent of epidermis; transition metal ion binding	Cell adhesion; epidermis morphogenesis; neutrophil degranulation
15	Mucin-16	Thought to provide a protective, lubricating barrier against particles and infectious agents at mucosal surfaces	O-glycan processing; cell adhesion; stimulatory C-type lectin receptor signaling pathway
16	Integrin alpha-1	Collagen binding; collagen binding involved in cell-matrix adhesion; metal ion binding; protein phosphatase binding; signaling receptor binding	Activation of MAPK activity; neuron projection morphogenesis; neutrophil chemotaxis; neuron apoptotic process; phosphoprotein phosphatase activity; vasodilation
17	Zinc-alpha-2-glycoprotein	Protein transmembrane transporter activity; ribonuclease activity	Cell adhesion; detection of chemical stimulus involved in sensory perception of bitter taste; retina homeostasis; transmembrane transport
18	Prelamin-A/C	Identical protein binding; structural molecule activity	Protein localization to nucleus and protein stability; cellular response to hypoxia; nuclear envelope organization; telomere maintenance
19	class I histocompatibility antigen, B-48 alpha chain	Peptide antigen binding	Antigen processing and presentation of exogenous peptide antigen via MHC class I, TAP-dependent; antigen processing and presentation of exogenous peptide antigen
20	Solute carrier family 40-member 1	Iron ion transmembrane transporter activity; peptide hormone binding	Cellular iron ion homeostasis; iron ion export across plasma membrane; iron ion transmembrane transport; lymphocyte homeostasis; spleen trabecula formation
21	Interferon-induced transmembrane protein 1	Inhibits the entry of viruses to the host cell cytoplasm, permitting endocytosis, but preventing subsequent viral fusion	Cell surface receptor signaling pathway; response to interferon alpha or beta; type I interferon signaling pathway
22	Proteasome subunit alpha type-7	Endopeptidase activity; identical protein binding; threonine-type endopeptidase activity	Ubiquitin dependent protein catabolic process; post translational protein modification; protein deubiquitination; protein polyubiquitination
23	Multifunctional protein ADE2	Cadherin binding; phosphoribosylaminoimidazole carboxylase synthase activity	Purine nucleobase biosynthetic process; purine ribonucleoside monophosphate biosynthetic process
24	Serine/threonine-protein phosphatase 2A 65 kDa regulatory subunit A alpha isoform	Protein antigen binding; protein heterodimerization activity; protein phosphatase regulator activity; protein serine/threonine phosphatase activity	RNA splicing; ceramide metabolic process; peptidyl serine dephosphorylation; protein dephosphorylation
25	Plexin-A4	Semaphorin receptor activity	Chemorepulsion of branchiomotor axon; regulation of axonogenesis, axon extension and GTPase activity; semaphorin-plexin signaling pathway involved in axon guidance
26	E3 ubiquitin-protein ligase TRIM58	Dynein heavy chain binding; dynein intermediate chain binding; ubiquitin protein ligase activity; zinc ion binding	Protein autoubiquitination; protein polyubiquitination; regulation of nuclear migration along microtubule; ubiquitin dependent protein catabolic process
27	Endoplasmic reticulum resident protein 44	Protein disulfide isomerase activity	Cell redox homeostasis; glycoprotein metabolic process; response to endoplasmic reticulum stress and unfolded protein

### GO analysis outcomes of the differentially expressed proteins

A GO analysis was employed to categorize the protein classification, molecular function, cellular composition, biological process, and mechanism.

#### GO analysis outcomes of the up-regulated proteins

**Figure 4:**
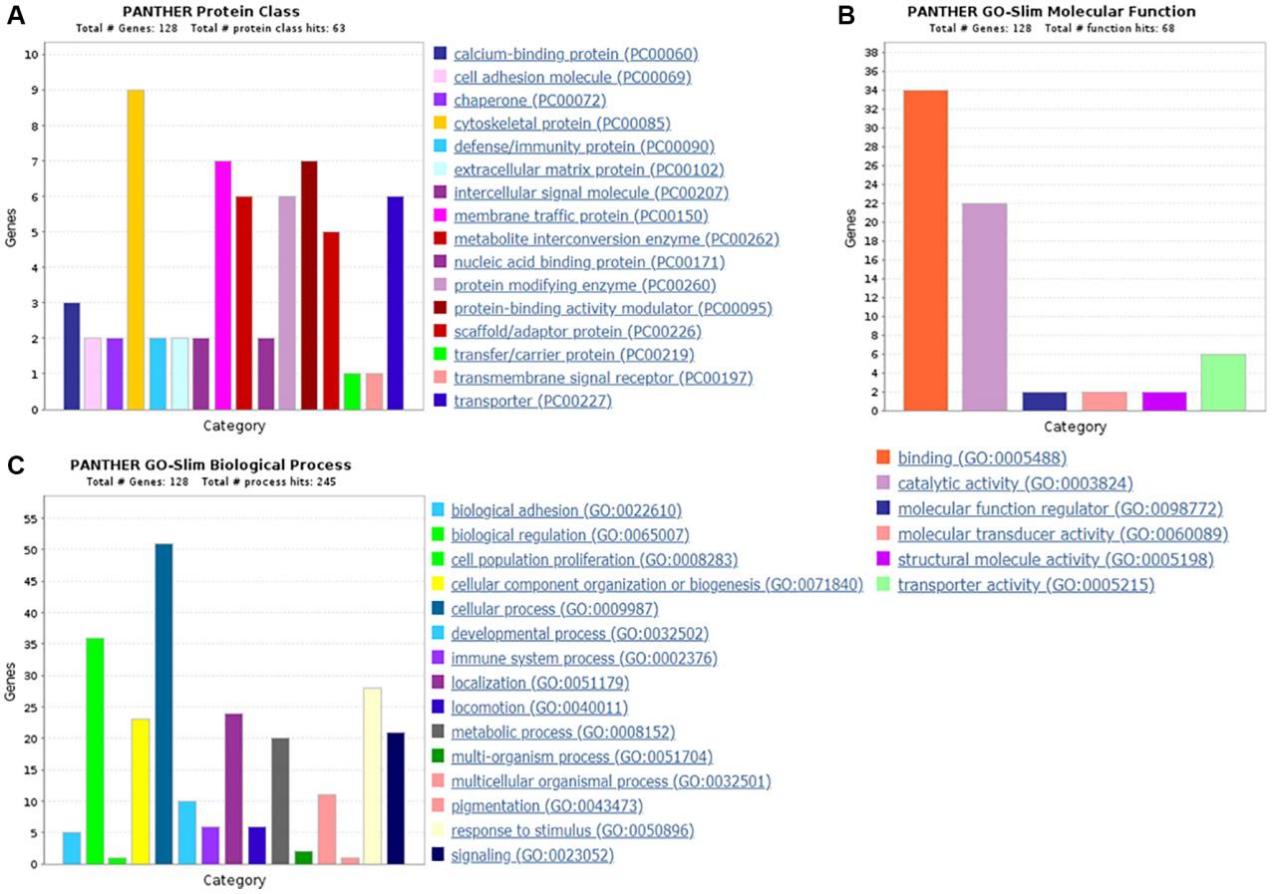
**The GO analysis results of up-regulated proteins in the PSCI group.** (**A**) The protein classification of up-regulated proteins. (**B**) The molecular function of up-regulated proteins. (**C**) The biological process of up-regulated proteins.

**Figure 5:**
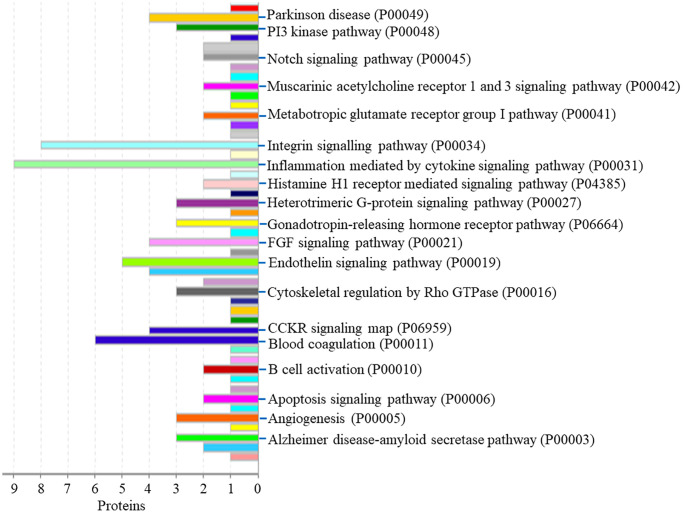
The pathway of up-regulated proteins in the PSCI group.

Protein classification results indicated that 7.0%, 5.5%, 4.7%, 4.7%, 5.5%, 3.9% and 4.7% of these 128 up-regulated proteins were cytoskeletal protein, membrane traffic protein, metabolite interconversion enzyme, protein modifying enzyme, protein-binding activity modulator, scaffold/adaptor protein, and transporter, respectively (Figure 4A). Molecular function classification results indicated that 26.6%, 17.2%, 4.7% of these 128 up-regulated proteins were binding, catalytic activity, and transporter activity, respectively (Figure 4B). In biological process classification, the majority of the proteins were identified to be contributed in biological regulation (28.1%), organization of cellular component or biogenesis (18.0%), cellular process (39.8%), localization (18.8%), metabolic process (15.6%), response to stimulus (21.9%), and signaling (16.4%), respectively (Figure 4C). Based on the categorization of pathways, most of these proteins were associated with blood coagulation (4.8%), CCKR signaling map (3.1%), EGF receptor signaling pathway (3.1%), endothelin signaling pathway (3.9%), FGF signaling pathway (3.1%), inflammation induced by chemokine and cytokine signaling pathway (7.0%), integrin signaling pathway (6.3%), PI3 kinase pathway (2.3%) (Figure 5). The blood coagulation pathway included proteinase-activated receptor 4, platelet glycoprotein V, integrin alpha-IIb, platelet glycoprotein Ib alpha chain, platelet glycoprotein Ib beta chain, and platelet glycoprotein IX. The endothelin signaling pathway included endothelin-converting enzyme 1, cAMP-dependent protein kinase catalytic subunit beta, protein kinase C beta type, mitogen-activated protein kinase 1, and guanine nucleotide-binding protein subunit alpha-14.

#### GO analysis outcomes of the down-regulated proteins

**Figure 6:**
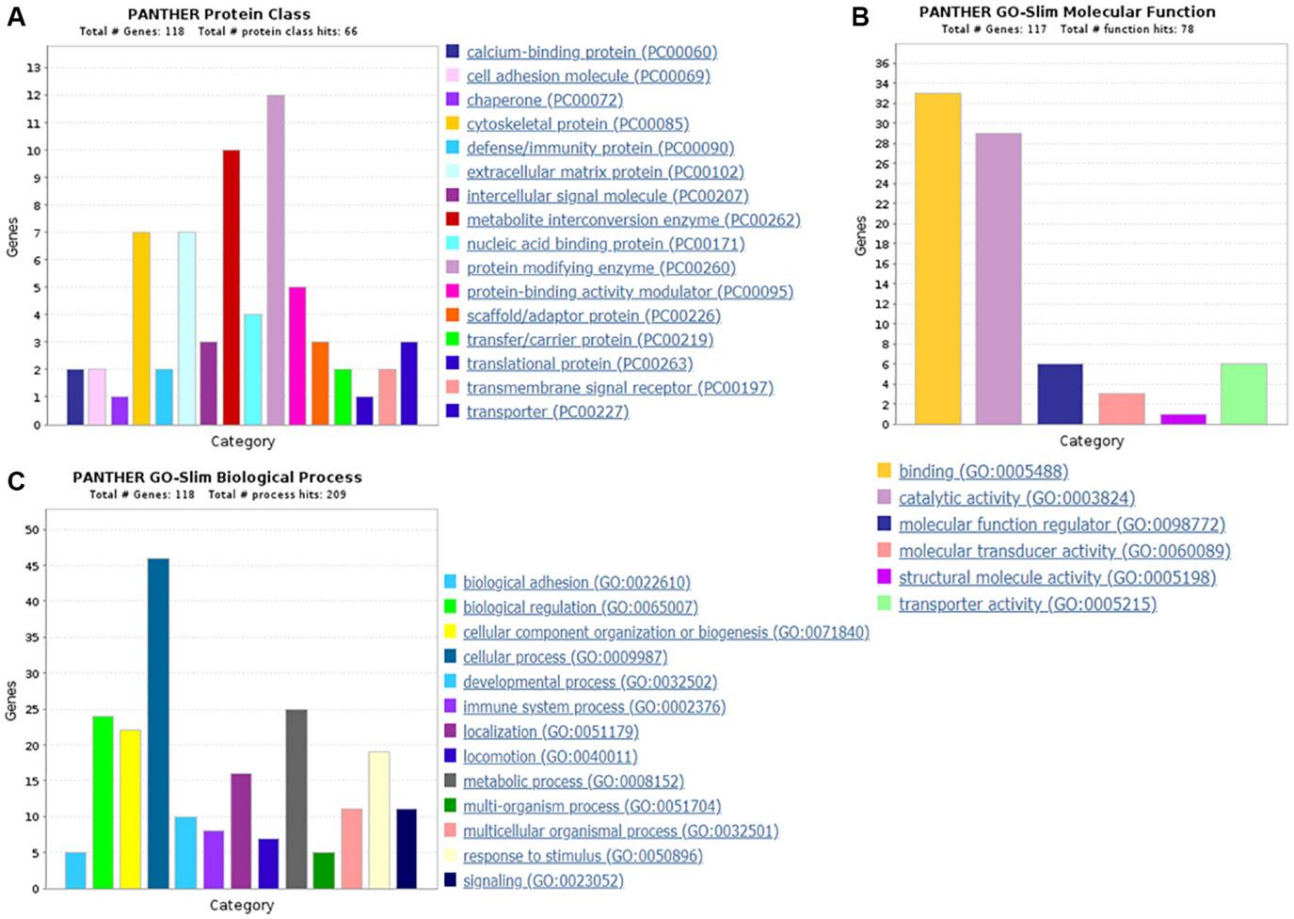
**The GO analysis results of down-regulated proteins in the PSCI group.** (**A**) The protein classification of down-regulated proteins. (**B**) The molecular function of down-regulated proteins. (**C**) The biological process of down-regulated proteins.

**Figure 7:**
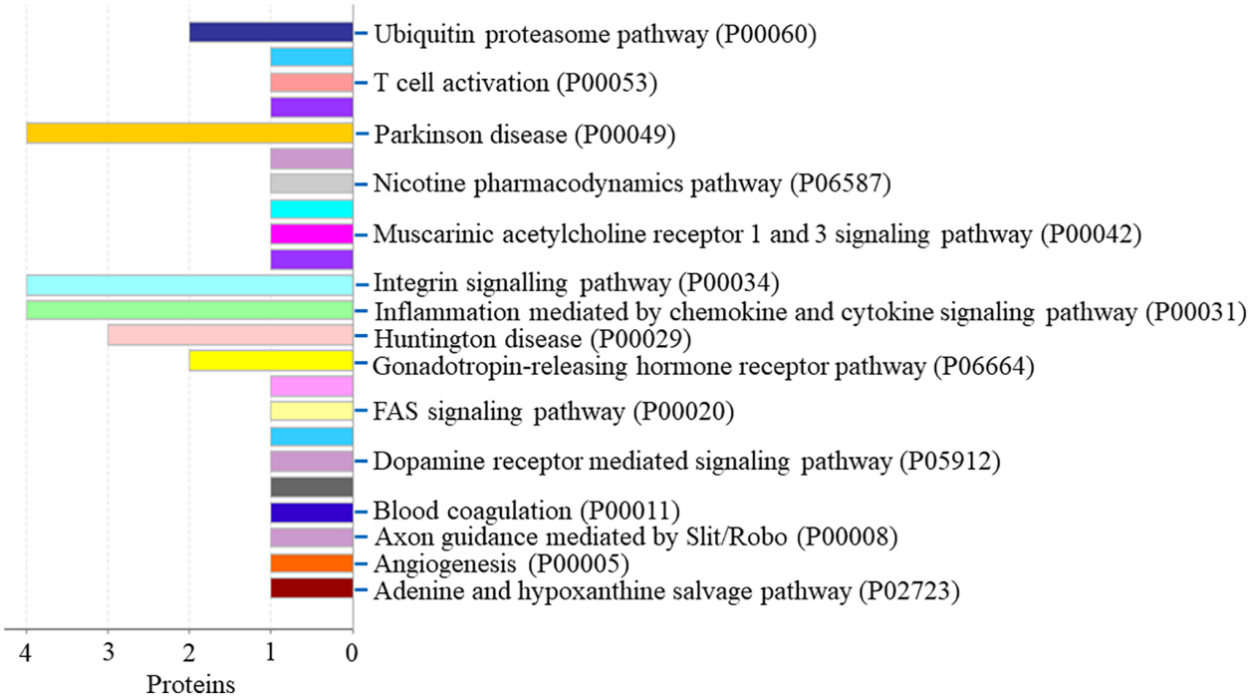
The pathway of down-regulated proteins in the PSCI group.

Protein classification results indicated that 5.9%, 5.9%, 8.5%, 3.4%, 10.2% and 4.2% of these 118 down-regulated proteins were cytoskeletal protein, extracellular matrix protein, metabolite interconversion enzyme, nucleic acid binding protein, protein modifying enzyme, and protein-binding activity modulator, respectively (Figure 6A). Molecular function classification results indicated that 28.2%, 24.8%, 5.1%, 2.6% and 5.1% of these down-regulated proteins were binding, catalytic activity, molecular function regulator, molecular transducer activity and transporter activity, respectively (Figure 6B). Throughout the biological process classification, the majority of the proteins were identified to be contributed in biological regulation (20.3%), cellular composition organization or biogenesis (18.6%), cellular process (39.0%), immune system process (6.8%), localization (13.6%), metabolic process (21.2%), multicellular organismal process (9.3%), response to stimulus (16.1%) and signaling (9.3%) (Figure 6C). Based on the categorization of pathways, most of these proteins were connected to gonadotropin-releasing hormone receptor pathway (1.7%) and ubiquitin proteasome pathway (1.7%) (Figure 7). The chemokine and cytokine signaling pathway which induced the inflammation included rho-related GTP-binding protein, platelet factor 4 variant, C5a anaphylatoxin chemotactic receptor 1, and C-X-C chemokine receptor type 2. The integrin signaling pathway included collagen alpha-1(I) chain, collagen alpha-2(I) chain, integrin alpha-1, and ADP-ribosylation factor 6.

### PPI networks analysis results

#### PPI networks analysis results of the up-regulated proteins

**Figure 8:**
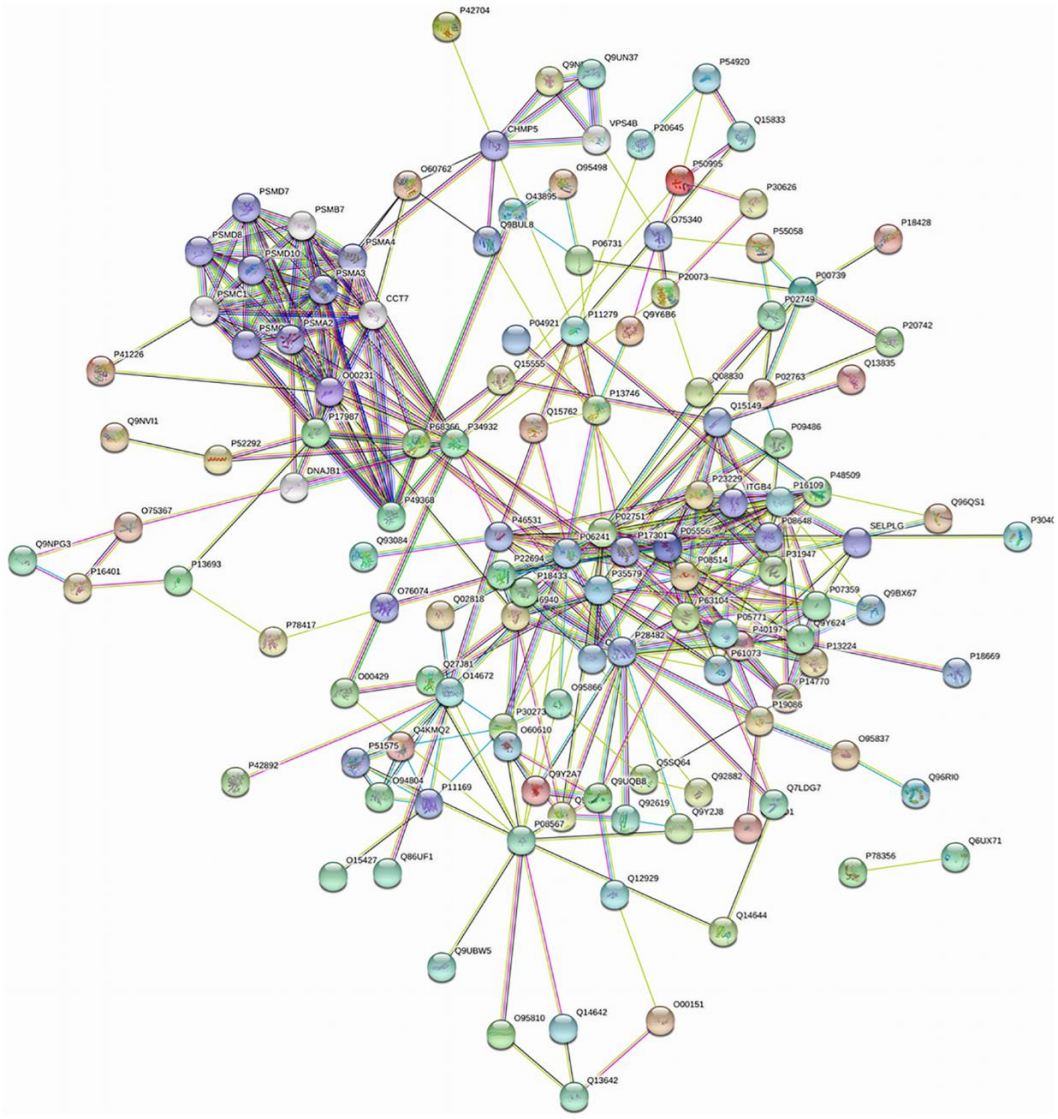
The PPI regulation network of up-regulated proteins in the PSCI group.

**Figure 9:**
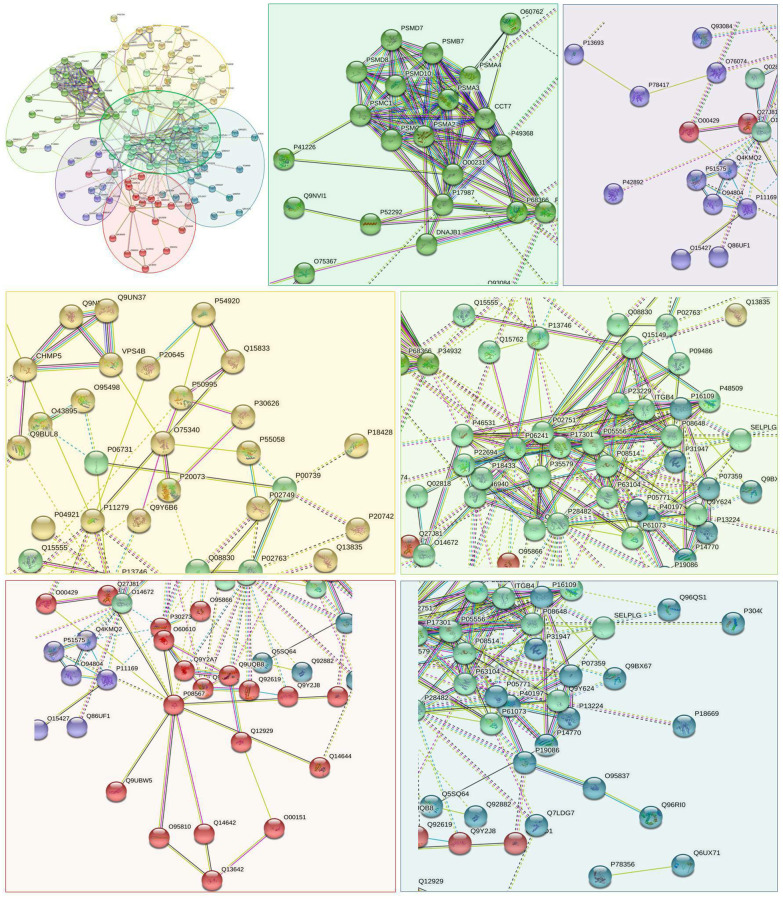
**The PPI network means clustering of up-regulated proteins in the PSCI group.** The PPI network is clustered to a specified number of clusters.

Utilizing STRING analysis, a controlled PPI network with high-quality was constructed. 127 up-regulated proteins were suitable for PPI network analysis (focus molecule) and PPI networks with high-quality were built according to the STRING database. A complete PPI regulation network with 127 up-regulated proteins were presented in Figure 8. The network clustering results showed that the PPI network consists of six specified function clusters that comprise proteins with similar functions and are expressed by various colors (Figure 9). These six visualized interaction function clusters (sub-networks) were related to degradation of ubiquitinated proteins and folding of proteins (lime green), calcium-dependent protein binding and ESCRT III complex disassembly (yellow), cytoskeleton reorganization and platelet aggregation (green), phospholipid scrambling of phosphatidylserine in platelets and ATP mediates synaptic transmission (purple), lipid binding, signal transduction (red), and blood coagulation, intrinsic pathway (blue), respectively. The PPI network which included the anticipated functional intermediate partners are presented in Table 7.

**Table 7:** The symbols and full names of the predicted functional intermediate partners in the PPI network of up-regulated expressed proteins shown in Figure 8.

**No.**	**Accession No.**	**Symbol**	**Full name**	**Molecular function**	**Number of amino acids**
1	P25787	PSMA2	Proteasome subunit alpha type-2	Component of the 20S core proteasome complex involved in the proteolytic degradation of most intracellular proteins.	234
2	P25789	PSMA4	proteasome subunit alpha type-4	Participates in the ATP-dependent degradation of ubiquitinated proteins and plays a key role in the maintenance of protein homeostasis by removing misfolded or damaged proteins.	261
3	P16144	ITGB4	Integrin beta-4	Binds to NRG1 (via EGF domain) and this binding is essential for NRG1-ERBB signaling	1822
4	P43686	PRS6B	26S proteasome regulatory subunit 6B	A multiprotein complex involved in the ATP-dependent degradation of ubiquitinated proteins.	418
5	P51665	PSMD7	26S proteasome non-ATPase regulatory subunit 7	Plays a key role in the maintenance of protein homeostasis by removing misfolded or damaged proteins.	324
6	O75832	PSMD10	26S proteasome non-ATPase regulatory subunit 10	Acts as a chaperone during the assembly of the 26S proteasome, specifically of the PA700/19S regulatory complex	226
7	Q9NZZ3	CHMP5	Charged multivesicular body protein 5	Peripherally associated component of the endosomal sorting required for transport complex III which is involved in multivesicular bodies formation and sorting.	219
8	Q14242	SELPLG	P-selectin glycoprotein ligand 1	A SLe(x)-type proteoglycan, which through high affinity, calcium-dependent interactions, mediates rapid rolling of leukocytes over vascular surfaces in inflammation.	428

#### PPI networks analysis results of the down-regulated proteins

**Figure 10:**
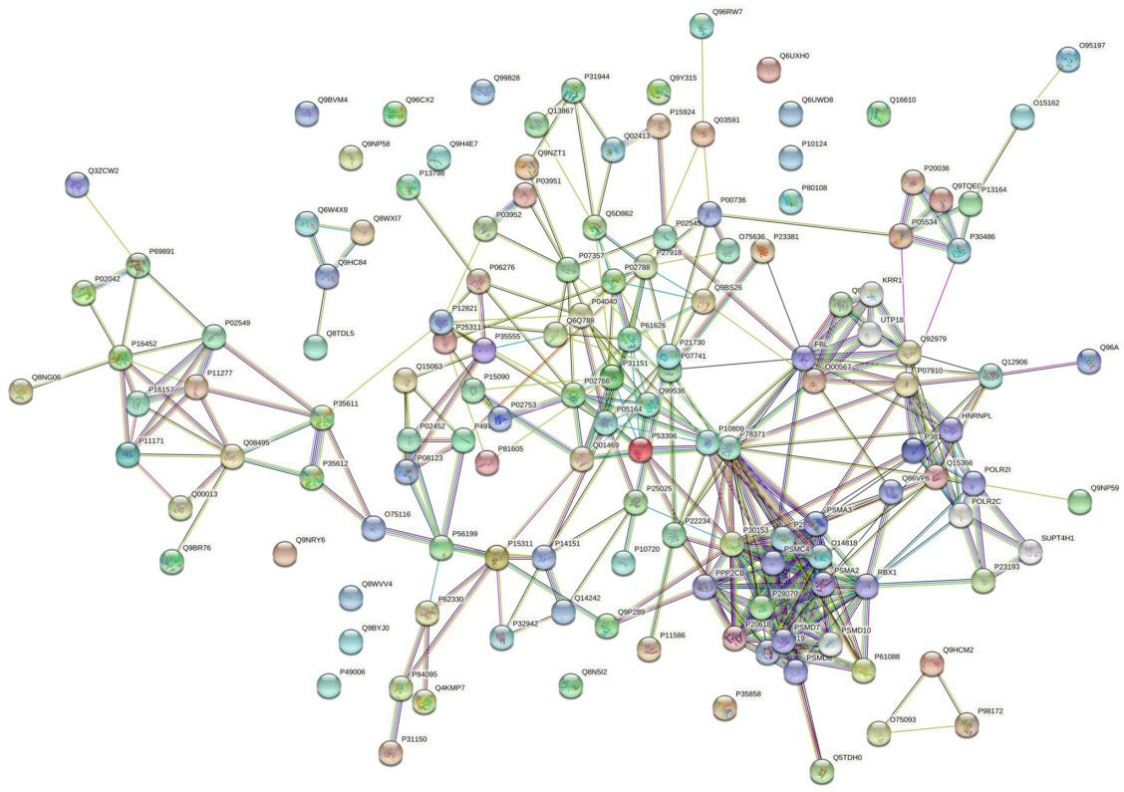
The PPI regulation network of down-regulated proteins in the PSCI group.

**Figure 11:**
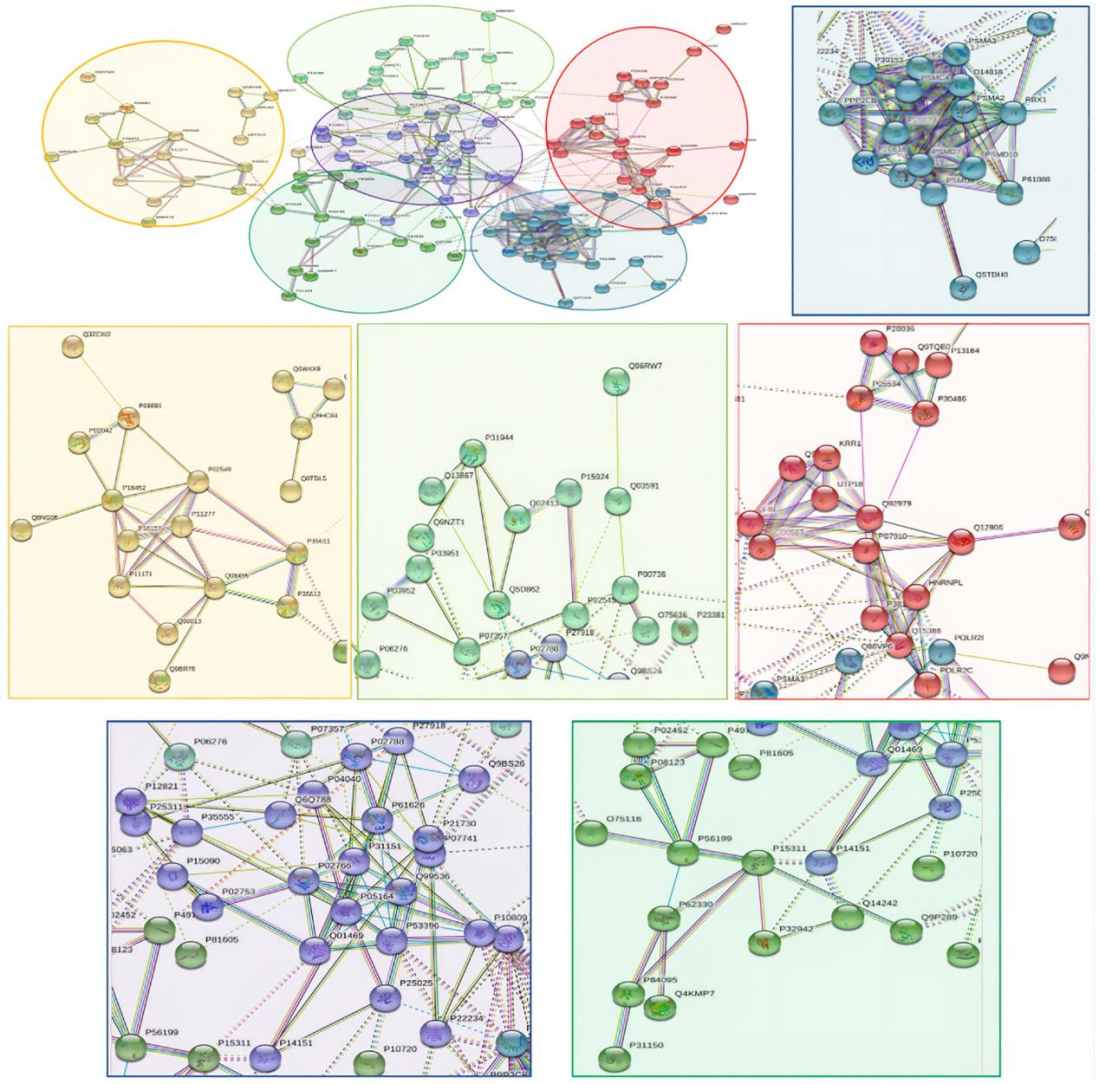
**The PPI network means clustering of down-regulated proteins in the PSCI group.** The PPI network is clustered to a specified number of clusters.

118 down-regulated proteins were suitable for PPI network analysis (focus molecule) and a controlled PPI networks with high-quality were constructed according to the STRING database. A complete regulation of PPI network by down-regulated proteins were presented in Figure 10. The network clustering result showed that the PPI network consists of six specified function clusters that comprise proteins with similar functions and are expressed by various colors (Figure 11). These six visualized interaction function clusters (sub-networks) were related to protein localization to juxtaparanode region of axon (yellow), cell adhesive protein binding, Fibrin Clot formation (green), mRNA splicing and RNA recognition (red), complement activation, lipid metabolism (purple), protein trafficking and cytoskeleton remodeling (lime green), and ATP-dependent degradation of ubiquitinated proteins (blue), respectively. The anticipated functional intermediate partners in the PPI network are presented in Table 8.

**Table 8:** The symbols and full names of the predicted functional intermediate partners in the PPI network of down-regulated expressed proteins shown in Figure 10.

**No.**	**Accession No.**	**Symbol**	**Full name**	**Molecular function**	**Number of amino acids**
1	P22087	FBL	rRNA 2′-O-methyltransferase fibrillarin	S-adenosyl-L-methionine-dependent methyltransferase that has the ability to methylate both RNAs and protein, catalyzing the site-specific 2′-hydroxyl methylation of ribose moieties in pre-ribosomal RNA.	321
2	P14866	HNRNPL	Heterogeneous nuclear ribonucleoprotein L	Splicing factor binding to exonic or intronic sites and acting as either an activator or repressor of exon inclusion, Exhibiting a binding preference for CA-rich elements.	589
3	P36954	POLR2I	DNA-directed RNA polymerase II subunit RPB9	DNA-dependent RNA polymerase catalyzes the transcription of DNA into RNA using the four ribonucleoside triphosphates as substrates.	125
4	P62714	PPP2CB	Serine/threonine-protein phosphatase 2A catalytic subunit beta isoform	PP2A can modulate the activity of phosphorylase B kinase casein kinase 2, mitogen-stimulated S6 kinase, and MAP-2 kinase.	309
5	P25788	PSMA3	Proteasome subunit alpha type-3	Plays numerous essential roles within the cell by associating with different regulatory particles.	255
6	P48556	PSMD8	26S proteasome non-ATPase regulatory subunit 8	Component of the 26S proteasome and participates in apoptosis or DNA damage repair.	350
7	P62877	RBX1	E3 ubiquitin-protein ligase RBX1	E3 ubiquitin ligase component of multiple cullin-RING- based E3 ubiquitin-protein ligase (CRLs) complexes which mediate the ubiquitination and subsequent proteasomal degradation of target proteins.	108

### ELISA quantitative determination results of plasma proteins

**Figure 12:**
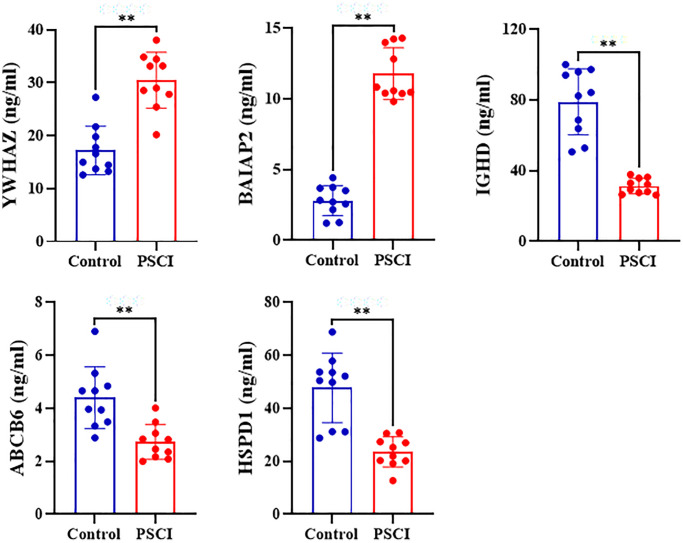
Plasma levels of human 14-3-3 protein zeta/delta (YWHAZ), human Brain-Specific Angiogenesis Inhibitor 1-Associated Protein 2 (BAIAP2), human IgD (IGHD), human ATP Binding Cassette Subfamily B Member 6, Mitochondrial (ABCB6), and human Heat Shock Protein 60 HSPD1 in patients with and without post stroke cognitive impairment.

Compared with control group, human 14-3-3 protein zeta/delta (YWHAZ) and human brain-specific angiogenesis suppressor 1 correlated protein 2 (BAIAP2) levels of plasma were significantly increased (*P* < 0.01) while that of human IgD (IGHD), human ATP binding cassette subfamily B member 6, mitochondrial (ABCB6) and human heat shock protein 60 (HSPD1) were significantly decreased in patients with and without PSCI (*P* < 0.01) (Figure 12).

## DISCUSSION

### General comments

To further explore the molecular mechanism of cognitive impairment, label-free quantitative proteomics were employed to analyse the differential expressed proteins of plasma exosome in PSCI patients. Proteomics identified 259 differentially expressed proteins, containing 131 upregulated proteins and 128 downregulated proteins. These upregulated proteins are connected to ubiquitinated proteins degradation, calcium dependent protein binding, reorganization of cytoskeleton and platelet aggregation and blood coagulation. These downregulated proteins are related to protein localization to juxtaparanode region of axon, cell adhesive protein binding, fibrin clot formation, complement activation, lipid metabolism and ATP-dependent degradation of ubiquitinated proteins. The mechanisms of cognitive impairment of PSCI are related to blood flow regulation, energy metabolism, protein folding and degradation, cell apoptosis, synaptic plasticity. These were discussed in detail below.

### Blood flow regulation associated proteins

Plasma kallikrein, a multifunctional serine protease associated with activation of contact coagulation [12]. Plasma kallikrein mechanisms of action can be utilized to support pro-thrombotic or anti-thrombotic characteristics. The kallikrein-kinin system suppresses thrombin-induced platelet activation, indicating an anti-thrombotic function [13]. Plasma kallikrein decreased collagen-induced platelet activation via binding collagen [14]. Whereas, the effect of pro-thrombotic is suggested by the plasma kallikrein critical role in contact activation by conversion of FXII to FXIIa. Additionally, plasma kallikrein converts prorenin to renin, which then converts angiotensinogen to angiotensin I [15]. Plasma kallikrein had been implicated in contributing to both hematoma expansion and thrombosis in stroke [16]. The outcomes of this investigation revealed that the plasma kallikrein expression was downregulated proteins. Plasma kallikrein may influence the occurrence and development of acute stroke through the activation and transformation pathway of FXII.

Platelet glycoprotein V (gpV), is a membrane constituent which containing an 82 kDa relative molecule mass, correlates with the leucine-rich proteins family. It is only expressed in platelets and megakaryocytes, and is non-covalently correlated with the gpIb-IX complex to develop a receptor for von Willebrand factor and thrombin [17, 18]. Hence, the GPIb-V-IX complex serve as the vWF receptor and modulates adhesion of vWF-dependent platelet to blood vessels. Platelet adhesion to damaged vascular surfaces in the arterial circulation is a crucial initiating event in hemostasis [19]. Platelet glycoprotein V may utilize as a platelet activation *in vivo* marker in thrombotic conditions. The expression of platelet-glycoprotein V in patients suffering from acute stroke is elevated, which is consistent with the study of Amin HM et al. [20]. Therefore, platelet glycoprotein V may play a protective role in the brain through blood coagulation. Fibrinogen like protein 1 (FGL1) is a released protein having mitogenic effect on primary hepatocytes. FGL1 includes an N-terminal signal recognition peptide, a potential N-terminal coil-coil domain, a C-terminal fibrinogen related domain (FReD) and multiple cysteines presumably utilized for inter and intra molecular disulfide bonds [21]. FGL1 may perform a potential function in these processes such as proliferation, angiogenesis, apoptosis and extracellular matrix modulation like structurally comparable proteins (angiopoietins, tenascins, fibrinogen) [22, 23]. Furthermore, the presence of FGL1 in the serum of rats after cytokine stimulation indicates that it could function as a significant biomarker for systemic inflammation [24]. Therefore, FGL1 may mediate the inflammatory response directly or indirectly in acute stroke. Our result of the increased expression of this protein just confirms this hypothesis.

### Energy metabolism

ATP-binding cassette sub-family B member 6 (ABCB6), a member of adenosine triphosphate–binding cassette (ABC) transporter the family. It binds with heme and porphyrins and have a function in their ATP-dependent uptake into the mitochondria and plays a crucial role in the synthesis of heme [25]. Some researches have found that ABCB6 expression is protective against various results elevating oxidative stress, including exposure of arsenite [26, 27]. The outcomes of this investigation revealed that the expression level of ABCB6 reduced in patients suffering from acute stroke. The ABCB6 expression and function of closely related to the oxidation mechanism of mitochondria [25]. Therefore, ABCB6 may serve a protective function in the brain via antioxidant mechanisms.

### Synaptic plasticity

Brain-specific angiogenesis inhibitor 1-associated protein 2 (BAIAP2), adapter protein that connects membrane-bound small G-proteins to cytoplasmic effector proteins. Subsequent researches have conclusively proven BAIAP2 serves as an essential regulator of membrane and actin dynamics at subcellular structures rich in actin, such as filopodia and lamellipodia [28–30]. Actin skeleton and its dynamics play an important role in the excitatory synaptic transmission and plasticity regulation [31, 32]. The results of this investigation revealed that the expression level of BAIAP2 increased in individuals with acute stroke. In the brain, BAIAP2 may perform a neuroprotective function by improving synaptic function.

## CONCLUSION

In conclusion, the present study found 259 differentially expressed proteins such as 131 upregulated proteins and 128 downregulated proteins using label-free quantitative proteomics approach in plasma exosome of PSCI patients. The findings suggested that the mechanism of cognitive impairment may be related to blood flow regulation, energy metabolism, protein folding and degradation, cell apoptosis, synaptic plasticity, stress response and protein phosphorylation in PSCI patients. Therefore, these proteins may be target-related proteins and shed light on pathogenesis mechanisms on a global scale of cognitive impairment at plasma exosome proteins level in PSCI patients. The disorders of plasma exosome proteomics may be explained the cognitive impairment in PSCI patients. Further association studies need to be clarified.

## Supplementary Materials

Supplementary Tables
