# Supplementary material for: Plasma exosome proteomics reveals the pathogenesis mechanism of post-stroke cognitive impairment
Source: Aging (Albany NY). 2023 May 20;15(10):4334–62. doi: 10.18632/aging.204738 (PMC10258006; doi:10.18632/aging.204738)
Supplement: Supplementary Tables [file aging-15-204738-s001.pdf]

SUPPLEMENTARY TABLES

Supplementary Table 1. The identification results of the other 101 up-regulated proteins between the PSCI and control groups.

No.	UniProt ID	Target protein	Gene symbol	Protein score	Mr (kDa)	Fold change (PSCI/ Control)	*P*-value
1	Q04828	Aldo-keto reductase family 1 member C1	AKR1C1	7.0284	36.788	4.13	<0.001
2	P50995	Annexin A11	ANXA11	55.951	54.389	3.55	<0.001
3	P20073	Annexin A7	ANXA7	60.69	52.739	4.08	<0.001
4	Q4KMQ2	Anoctamin-6	ANO6	16.419	106.16	2.94	0.018
5	Q96QS1	Tetraspanin-32	TSPAN32	17.05	34.63	6.05	<0.001
6	O75882	Attractin	ATRN	5.3994	158.54	2.06	0.008
7	P55957	BH3-interacting domain death agonist	BID	5.4515	21.994	3.07	0.001
8	Q9UBW5	Bridging integrator 2	BIN2	16.654	61.874	5.65	<0.001
9	Q9Y376	Calcium-binding protein 39	CAB39	6.2081	39.869	13.75	<0.001
10	P22694	cAMP-dependent protein kinase catalytic subunit beta	PRKACB	15.569	40.622	2.84	<0.001
11	P06731	Carcinoembryonic antigen-related cell adhesion molecule 5	CEACAM5	3.4781	76.794	5.52	0.001
12	P20645	Cation-dependent mannose-6-phosphate receptor	M6PR	4.1554	30.993	2.10	<0.001
13	P48509	CD151 antigen	CD151	22.583	28.295	2.24	<0.001
14	Q96DZ9	CKLF-like MARVEL transmembrane domain-containing protein 5	CMTM5	8.2909	24.652	8.11	<0.001
15	P30447	Class I histocompatibility antigen, A-23 alpha chain	HLA-A	17.273	40.732	11.92	<0.001
16	Q96FN4	Copine-2	CPNE2	4.6578	61.189	2.08	<0.001
17	O75367	Core histone macro-H2A.1	H2AFY	5.632	39.617	2.09	<0.001
18	P61073	C-X-C chemokine receptor type 4	CXCR4	10.04	39.745	11.32	<0.001
19	Q9Y4D1	Disheveled-associated activator of morphogenesis 1	DAAM1	25.077	123.47	3.47	0.032
20	O14672	Disintegrin and metalloproteinase domain-containing protein 10	ADAM10	87.302	84.141	2.26	<0.001
21	O60762	Dolichol-phosphate mannosyltransferase subunit 1	DPM1	3.0248	29.634	4.54	<0.001
22	O00429	Dynamin-1-like protein	DNM1L	4.8548	81.876	3.84	<0.001
23	P42892	Endothelin-converting enzyme 1	ECE1	8.9951	87.163	10.28	<0.001
24	Q12929	Epidermal growth factor receptor kinase substrate 8	EPS8	18.03	91.88	7.17	<0.001
25	Q96PL5	Erythroid membrane-associated protein	ERMAP	6.8807	52.604	3.93	<0.001
26	Q9NVI1	Fanconi anemia group I protein	FANCI	2.4495	149.32	4.29	<0.001
27	Q15149	Plectin	PLEC	19.19	531.78	2.12	0.003
28	Q86WI1	Fibrocystin-L	PKHD1L1	8.8806	465.73	3.29	<0.001
29	P02751	Fibronectin	FN1	323.31	262.62	2.03	<0.001
30	P19086	Guanine nucleotide-binding protein G(z) subunit alpha	GNAZ	36.541	40.923	4.24	<0.001
31	O95837	Guanine nucleotide-binding protein subunit alpha-14	GNA14	6.3116	41.57	3.93	<0.001
32	P51790	H(+)/Cl(−) exchange transporter 3	CLCN3	4.0367	90.965	6.38	<0.001
33	P00739	Haptoglobin-related protein	HPR	125.79	39.029	2.38	<0.001
34	P80422	Ig gamma lambda chain V-II region DOT		2.8868	11.787	3.33	0.018
35	P01604	Ig kappa chain V-I region Kue		3.2276	12.126	9.77	<0.001
36	P01610	Ig kappa chain V-I region WEA		4.7707	11.84	10.45	0.047
37	P18135	Ig kappa chain V-III region HAH		9.7585	14.073	12.72	0.002
38	P06311	Ig kappa chain V-III region IARC/BL41		27.922	14.07	22.59	0.001
39	P06889	Ig lambda chain V-IV region MOL		10.976	11.143	26.71	<0.001
40	P52292	Importin subunit alpha-1	KPNA2	5.1473	57.861	8.02	<0.001
41	P17301	Integrin alpha-2	ITGA2	93.857	129.29	2.26	<0.001
42	P23229	Integrin alpha-6	ITGA6	165.92	126.6	2.70	<0.001

43	P08514	Integrin alpha-IIb	ITGA2B	323.31	113.38	2.59	<0.001
44	P05556	Integrin beta-1	ITGB1	93.303	88.414	2.02	<0.001
45	Q27J81	Inverted formin-2	INF2	10.723	135.62	2.59	<0.001
46	Q9Y624	Junctional adhesion molecule A	F11R	37.373	32.583	3.10	<0.001
47	Q9BX67	Junctional adhesion molecule C	JAM3	7.2824	35.02	3.12	<0.001
48	P42704	Leucine-rich PPR motif-containing protein, mitochondrial	LRPPRC	2.5029	157.9	2.34	0.046
49	Q6ZUX7	Lipoma HMGIC fusion partner-like 2 proteins	LHFPL2	5.4873	24.486	16.11	<0.001
50	P18428	Lipopolysaccharide-binding protein	LBP	68.595	53.383	3.19	<0.001
51	Q5SQ64	Lymphocyte antigen 6 complex locus protein G6f	LY6G6F	9.1082	32.464	2.39	<0.001
52	P11279	Lysosome-associated membrane glycoprotein 1	LAMP1	8.3269	44.882	7.21	<0.001
53	Q15555	Microtubule-associated protein RP/EB family member 2	MAPRE2	7.2936	37.031	4.92	0.005
54	Q92619	Minor histocompatibility protein HA-1	HMHA1	9.453	124.61	10.33	<0.001
55	P28482	Mitogen-activated protein kinase 1	MAPK1	2.6431	41.389	2.53	0.016
56	O15427	Monocarboxylate transporter 4	SLC16A3	7.7629	49.469	4.83	0.001
57	Q15746	Myosin light chain kinase, smooth muscle	MYLK	8.0124	210.71	2.43	0.036
58	Q9Y2A7	Nck-associated protein 1	NCKAP1	18.888	128.79	2.21	<0.001
59	P35579	Myosin-9	MYH9	323.31	226.53	2.20	<0.001
60	Q02818	Nucleobindin-1	NUCB1	10.379	53.879	3.93	<0.001
61	Q92882	Osteoclast-stimulating factor 1	OSTF1	4.9459	23.787	2.49	<0.001
62	O00151	PDZ and LIM domain protein 1	PDLIM1	22.463	36.071	2.59	0.008
63	P78356	Phosphatidylinositol 5-phosphate 4-kinase type-2 beta	PIP4K2B	3.3849	47.377	2.61	<0.001
64	P18669	Phosphoglycerate mutase 1	PGAM1	4.9925	28.804	4.60	0.016
65	Q13835	Plakophilin-1	PKP1	3.0107	82.86	15.11	<0.001
66	P07359	Platelet glycoprotein Ib alpha chain	GP1BA	72.749	71.539	2.30	<0.001
67	P13224	Platelet glycoprotein Ib beta chain	GP1BB	34.808	21.717	2.50	<0.001
68	P08567	Pleckstrin	PLEK	96.786	40.124	3.68	<0.001
69	Q6UX71	Plexin domain-containing protein 2	PLXDC2	3.1051	59.582	5.86	<0.001
70	Q9BUL8	Programmed cell death protein 10	PDCD10	6.8355	24.701	2.55	0.049
71	O75340	Programmed cell death protein 6	PDCD6	11.365	21.868	2.14	<0.001
72	O00231	proteasome non-ATPase regulatory subunit 11	PSMD11	2.9363	47.463	2.14	0.002
73	O60610	Protein diaphanous homolog 1	DIAPH1	24.691	141.35	2.72	<0.001
74	O95866	Protein G6b	G6B	27.188	26.163	3.24	<0.001
75	P05771	Protein kinase C beta type	PRKCB	16.466	76.868	3.58	<0.001
76	Q9Y2J8	Protein-arginine deiminase type-2	PADI2	4.2012	75.563	2.30	0.004
77	Q96RI0	Proteinase-activated receptor 4	F2RL3	3.8785	41.133	5.32	<0.001
78	P16109	P-selectin	SELP	28.054	90.833	2.07	0.050
79	Q14644	Ras GTPase-activating protein 3	RASA3	65.234	95.698	2.73	<0.001
80	P46940	Ras GTPase-activating-like protein IQGAP1	IQGAP1	14.068	189.25	3.83	0.026
81	Q7LDG7	RAS guanyl-releasing protein 2	RASGRP2	13.93	69.248	2.51	<0.001
82	P18433	Receptor-type tyrosine-protein phosphatase alpha	PTPRA	11.171	90.599	2.12	0.017
83	Q9HBH0	Rho-related GTP-binding protein RhoF	RHOF	5.2317	23.625	4.04	<0.001
84	Q9BRU9	rRNA-processing protein UTP23 homolog	UTP23	2.6882	28.402	23.83	0.011
85	Q9NUV7	Serine palmitoyltransferase 3	SPTLC3	2.7378	62.049	2.30	<0.001
86	O95810	Serum deprivation-response protein	SDPR	59.51	47.173	2.86	<0.001
87	A6NMB1	Sialic acid-binding Ig-like lectin 16	SIGLEC16	2.8659	52.991	2.17	<0.001
88	P30626	Sorcin	SRI	14.391	21.676	5.40	<0.001
89	P09486	SPARC	SPARC	14.135	34.632	2.05	0.011
90	Q15833	Syntaxin-binding protein 2	STXBP2	94.853	66.452	2.94	<0.001
91	P17987	T-complex protein 1 subunit alpha	TCP1	9.5532	60.343	2.33	<0.001
92	P49368	T-complex protein 1 subunit gamma	CCT3	20.31	60.533	4.59	<0.001
93	Q86UF1	Tetraspanin-33	TSPAN33	23.298	31.538	3.87	<0.001

94	P13693	Translationally-controlled tumor protein	TPT1	2.975	19.595	2.85	0.005
95	P30408	Transmembrane 4 L6 family member 1	TM4SF1	3.2213	21.632	3.63	<0.001
96	P68366	Tubulin alpha-4A chain	TUBA4A	14.7	49.924	5.38	<0.001
77	Q14642	Type I inositol 1,4,5-trisphosphate 5-phosphatase	INPP5A	3.5125	47.819	2.14	<0.001
98	P06241	Tyrosine-protein kinase Fyn	FYN	4.4482	60.761	2.35	<0.001
99	Q9NPG3	Ubinuclein-1	UBN1	2.7915	121.52	4.40	0.002
100	P41226	Ubiquitin-like modifier-activating enzyme 7	UBA7	4.9628	111.69	5.16	<0.001
101	O95498	Vascular non-inflammatory molecule 2	VNN2	7.7606	58.502	2.10	0.024

In the title line, Exp. Mr represented the experimental molecular weight of the proteins.

Supplementary Table 2. The identification results of other 98 down-regulated proteins between the PSCI and control groups.

No.	UniProt ID	Target protein	Gene Symbol	Protein Score	Mr (kDa)	Fold change (PSCI/ Control)	*P*-value
1	Q00013	55 kDa erythrocyte membrane protein	MPP1	29.003	52.296	0.23	<0.001
2	P13798	Acylamino-acid-releasing enzyme	APEH	25.707	81.224	0.39	<0.001
3	P07741	Adenine phosphoribosyltransferase	APRT	5.640	19.608	0.23	<0.001
4	P35611	Alpha-adducin	ADD1	64.705	80.954	0.12	<0.001
5	P12821	Angiotensin-converting enzyme	ACE	47.608	149.710	0.45	<0.001
6	P16157	Ankyrin-1	ANK1	323.310	206.260	0.45	<0.001
7	Q6Q788	Apolipoprotein A-V	APOA5	26.542	41.212	0.38	<0.001
8	Q8N5I2	Arrestin domain-containing protein 1	ARRDC1	5.041	45.981	0.19	<0.001
9	P35612	Beta-adducin	ADD2	46.085	80.853	0.18	<0.001
10	Q13867	Bleomycin hydrolase	BLMH	3.605	52.562	0.15	<0.001
11	Q8TDL5	BPI fold-containing family B member 1	BPIFB1	11.262	52.441	0.05	<0.001
12	Q96CX2	BTB/POZ domain-containing protein KCTD12	KCTD12	6.607	35.700	0.12	<0.001
13	P11586	C-1-tetrahydrofolate synthase, cytoplasmic	MTHFD1	6.445	101.560	0.08	<0.001
14	P21730	C5a anaphylatoxin chemotactic receptor 1	C5AR1	13.851	39.335	0.36	<0.001
15	Q9NZT1	Calmodulin-like protein 5	CALML5	4.879	15.892	0.37	0.001
16	P49747	Cartilage oligomeric matrix protein	COMP	16.663	82.860	0.20	0.006
17	P31944	Caspase-14	CASP14	12.773	27.679	0.41	<0.001
18	P04040	Catalase	CAT	158.240	59.755	0.46	<0.001
19	Q9TQE0	Class II histocompatibility antigen, DRB1-9 beta chain	HLA-DRB1	35.992	29.826	0.41	<0.001
20	P03951	Coagulation factor XI	F11	28.429	70.108	0.39	<0.001
21	P02452	Collagen alpha-1(I) chain	COL1A1	3.211	138.940	0.13	<0.001
22	P08123	Collagen alpha-2(I) chain	COL1A2	6.514	129.310	0.47	<0.001
23	P00736	Complement C1r subcomponent	C1R	87.215	80.118	0.45	<0.001
24	Q03591	Complement factor H-related protein 1	CFHR1	3.435	37.650	0.46	<0.001
25	Q9BR76	Coronin-1B	CORO1B	3.423	54.234	0.40	<0.001
26	Q86VP6	Cullin-associated NEDD8-dissociated protein 1	CAND1	7.461	136.370	0.45	<0.001
27	P25025	C-X-C chemokine receptor type 2	CXCR2	5.076	40.759	0.19	0.001
28	Q08495	Dematin	DMTN	50.325	45.514	0.38	<0.001
29	Q9Y315	Deoxyribose-phosphate aldolase	DERA	11.727	35.230	0.31	0.001
30	P81605	Dermcidin	DCD	8.586	11.284	0.39	<0.001
31	Q02413	Desmoglein-1	DSG1	14.071	113.750	0.14	<0.001
32	P15924	Desmoplakin	DSP	107.470	331.770	0.02	<0.001
33	Q9H4E7	Differentially expressed in FDCP 6 homolog	DEF6	6.243	73.910	0.10	<0.001
34	P98172	Ephrin-B1	EFNB1	7.399	38.006	0.33	<0.001

35	P16452	Erythrocyte membrane protein band 4.2	EPB42	211.300	77.008	0.46	<0.001
36	Q16610	Extracellular matrix protein 1	ECM1	10.391	60.673	0.47	0.024
37	P15311	Ezrin	EZR	36.780	69.412	0.38	<0.001
38	P15090	Fatty acid-binding protein, adipocyte	FABP4	9.663	14.719	0.44	0.001
39	Q01469	Fatty acid-binding protein, epidermal	FABP5	13.170	15.164	0.40	<0.001
40	P35555	Fibrillin-1	FBN1	20.479	312.240	0.25	<0.001
41	Q9BYJ0	Fibroblast growth factor-binding protein 2	FGFBP2	6.010	24.581	0.34	<0.001
42	O75636	Ficolin-3	FCN3	130.590	32.903	0.48	<0.001
43	Q3ZCW2	Galectin-related protein	LGALSL	3.739	18.986	0.31	0.024
44	Q9BVM4	Gamma-glutamylaminecyclotransferase	GGACT	2.631	17.328	0.14	<0.001
45	Q96RW7	Hemicentin-1	HMCN1	9.912	613.380	0.25	<0.001
46	P02042	Hemoglobin subunit delta	HBD	42.271	16.055	0.28	<0.001
47	P69891	Hemoglobin subunit gamma-1	HBG1	11.663	16.140	0.33	<0.001
48	P07910	Heterogeneous nuclear ribonucleoproteins C1/C2	HNRNPCL4	11.389	33.670	0.11	<0.001
49	P05534	HLA class I histocompatibility antigen, A-24 alpha chain	HLA-A	57.269	40.688	0.49	0.003
50	P30450	HLA class I histocompatibility antigen, A-26 alpha chain	HLA-A	57.588	41.061	0.22	<0.001
51	P20036	HLA class II histocompatibility antigen, DP alpha 1 chain	HLA-DPA1	2.810	29.380	0.14	<0.001
52	P01621	Ig kappa chain V-III region NG9 (Fragment)		2.595	10.729	0.02	<0.001
53	P04209	Ig lambda chain V-II region NIG-84		3.539	11.581	0.50	<0.001
54	P35858	Insulin-like growth factor-binding protein complex acid labile subunit	IGFALS	10.043	66.034	0.34	<0.001
55	P32942	Intercellular adhesion molecule 3	ICAM3	11.777	59.540	0.31	<0.001
56	Q12906	Interleukin enhancer-binding factor 3	ILF3	3.242	95.337	0.32	<0.001
57	P02788	Lactotransferrin	LTF	43.636	78.181	0.31	<0.001
58	Q96AG4	Leucine-rich repeat-containing protein 59	LRRC59	5.097	34.930	0.49	0.001
59	P14151	L-selectin	SELL	34.681	42.187	0.29	<0.001
60	P61626	Lysozyme C	LYZ	7.020	16.537	0.22	<0.001
61	P49006	MARCKS-related protein	MARCKSL1	9.031	19.529	0.24	<0.001
62	Q02817	Mucin-2	MUC2	64.496	540.290	0.36	<0.001
63	Q9HC84	Mucin-5B	MUC5B	238.930	596.330	0.20	<0.001
64	Q6W4X9	Mucin-6	MUC6	9.907	257.050	0.26	0.002
65	P05164	Myeloperoxidase	MPO	5.226	83.868	0.10	<0.001
66	O00567	Nucleolar protein 56	NOP56	7.993	66.049	0.40	<0.001
67	Q15063	Periostin	POSTN	6.397	93.313	0.12	<0.001
68	P80108	Phosphatidylinositol-glycan-specific phospholipase D	GPLD1	4.472	92.335	0.33	<0.001
69	Q9NRY6	Phospholipid scramblase 3	PLSCR3	7.649	31.648	0.23	<0.001
70	P10720	Platelet factor 4 variant	PF4V1	7.572	11.553	0.26	<0.001
71	Q15366	Poly(rC)-binding protein 2	PCBP2	16.973	38.580	0.19	<0.001
72	Q9Y2R4	Probable ATP-dependent RNA helicase DDX52	DDX52	3.420	67.497	0.23	<0.001
73	P27918	Properdin	CFP	34.547	51.276	0.46	<0.001
74	P25789	Proteasome subunit alpha type-4	PSMA4	8.810	29.483	0.42	<0.001
75	P28070	Proteasome subunit beta type-4	PSMB4	5.199	29.204	0.48	<0.001
76	P11171	Protein 4.1	EPB41	192.910	97.016	0.30	<0.001
77	Q5TDH0	Protein DDI1 homolog 2	DDI2	4.051	44.522	0.49	<0.001
78	Q8WVV4	Protein POF1B	POF1B	21.398	68.064	0.38	0.002
79	P31151	Protein S100-A7	S100A7	3.630	11.471	0.39	<0.001

80	Q14242	P-selectin glycoprotein ligand 1	SELPLG	12.039	43.201	0.27	<0.001
81	P31150	Rab GDP dissociation inhibitor alpha	GDI1	15.814	50.582	0.35	<0.001
82	O95197	Reticulon-3	RTN3	3.396	112.610	0.43	0.001
83	P02753	Retinol-binding protein 4	RBP4	12.398	23.010	0.42	<0.001
84	O75116	Rho-associated protein kinase 2	ROCK2	10.343	160.900	0.19	<0.001
85	P84095	Rho-related GTP-binding protein	RHOG	24.350	21.308	0.30	<0.001
86	Q92979	Ribosomal RNA small subunit methyltransferase NEP1	EMG1	3.192	26.720	0.42	0.014
87	P38159	RNA-binding motif protein, X chromosome	RBMX	6.121	42.331	0.48	0.004
88	P10124	Serglycin	SRGN	13.192	17.652	0.28	<0.001
89	O75093	Slit homolog 1 protein	SLIT1	3.174	167.920	0.49	0.005
90	P02549	Spectrin alpha chain, erythrocytic 1	SPTA1	323.310	280.010	0.34	<0.001
91	P11277	Spectrin beta chain, erythrocytic	SPTB	323.310	246.470	0.32	<0.001
92	Q4KMP7	TBC1 domain family member 10B	TBC1D10B	2.593	87.198	0.41	0.001
93	P78371	T-complex protein 1 subunit beta	CCT2	27.382	57.488	0.34	<0.001
94	P23193	Transcription elongation factor A protein 1	TCEA1	35.693	33.969	0.31	0.021
95	Q6UWD8	Transmembrane protein C16orf54	C16orf54	24.211	24.359	0.44	<0.001
76	P02766	Transthyretin	TTR	4.849	15.887	0.46	<0.001
97	P23381	Tryptophan-tRNA ligase, cytoplasmic	WARS	5.162	53.165	0.30	<0.001
98	P61088	Ubiquitin-conjugating enzyme E2 N	UBE2N	3.957	17.138	0.22	<0.001

In the title line, Exp. Mr represented the experimental molecular weight of the proteins.
